# Assessment of the influence of vitamin D in patients with sepsis: a systematic review and meta-analysis

**DOI:** 10.3389/fnut.2025.1670083

**Published:** 2025-10-13

**Authors:** Hangqi Zhu, Keyi Li, Yulu Zhao, Juan Qin, Guolin Song

**Affiliations:** ^1^Department of Emergency Medicine, The Second Affiliated Hospital of Guizhou University of Traditional Chinese Medicine, Guiyang, Guizhou, China; ^2^Second School of Clinical Medicine, Guizhou University of Traditional Chinese Medicine, Guiyang, Guizhou, China; ^3^Department of Gynecology, Guiyang Maternal and Child Health Care Hospital, Guiyang, Guizhou, China

**Keywords:** sepsis, vitamin D, meta-analysis, clinical outcomes, pediatric and adult patients

## Abstract

**Background:**

This systematic review and meta-analysis seeks to extensively estimate the interrelation between vitamin D (VD) and clinical results among both pediatric and adult sepsis patients.

**Methods:**

A search was implemented through four databases (PubMed, Embase, Cochrane Library, and Web of Science) up to February 2025. Meta-analysis was implemented utilizing Stata 15 and Meta-Disc software.

**Results:**

Thirty-nine studies were included, encompassing 1,208 pediatric and neonatal sepsis patients, and 60,566 adult sepsis patients. The results showed that the average VD level in neonates with sepsis was 12.99 (95% CI: 8.11, 17.87), and the average VD level in children was 24.84 (95% CI: 21.34, 28.33). Their VD levels were considerably lower relative to healthy individuals or those without sepsis, with statistical distinction (*p* < 0.05). The aggregated prevalence of VD deficiency and insufficiency was 54%. When VD levels were <30 ng/mL, the aggregate prevalence of deficiency and insufficiency was the highest at 76%. A considerable interrelation between VD deficiency and mortality was identified, contrasted with the control group (*p* < 0.05). Among adults with sepsis, the average VD level was 17.12 (95% CI: 14.19, 20.05). Relative to the healthy cohort, VD levels substantially declined, with statistical distinction (*p* < 0.05); relative to those without sepsis, there was no statistical distinction in VD levels (*p* = 0.05). The pooled prevalence of VD deficiency and insufficiency was 55%. The deficiency of VD was considerably correlated with both the incidence and mortality of sepsis (*p* < 0.001). Supplementation with VD did not reduce the length of ICU stay (*p* = 0.67), but it can considerably reduce the risk of death (*p* < 0.05). The sensitivity and specificity of VD to forecast mortality among adult sepsis patients were 81 and 31%, respectively.

**Conclusion:**

Vitamin D status in both pediatric and adult sepsis individuals was predominantly in a deficient state, and the prevalence of VD deficiency and insufficiency is relatively high. VD deficiency was considerably linked to elevated mortality among pediatric sepsis individuals and also the incidence and mortality of adult sepsis individuals. VD may serve as a valuable biomarker to forecast mortality among adult sepsis individuals.

**Systematic review registration:**

https://www.crd.york.ac.uk/PROSPERO/view/CRD420250651346, CRD420250651346

## 1 Introduction

Sepsis is featured as a fatal organ failure originating from a dysregulated immune response to infection, which induces a systemic hyperinflammatory state. Severe sepsis can progress to septic shock, which is linked to an in-hospital mortality exceeding 40% (1). Sepsis and septic shock pose a serious global health threat and are principal causes of demise in intensive care units (ICU) (2). Recent data show that approximately 11 million deaths occur annually due to sepsis, accounting for nearly 20% of global mortality (3). The Surviving Sepsis Campaign Guidelines (SSCG) and the Japanese Sepsis Society clinical guidelines (J-SSCG) are high-impact guidelines established in recent years. Both of them advocate for early detection, effective control of infection sources, appropriate antimicrobial treatment, and adequate organ assistance to mitigate the burden of sepsis (4, 5). Therefore, it’s urgent to identify effective biomarkers and immune modulatory treatments to estimate the effect on the early diagnosis, therapeutic intervention, and prognosis of sepsis patients.

Recently, vitamin D (VD) has garnered particular attention for its crucial function in the immune system. VD can protect cells from harmful signals by suppressing inflammatory responses (6). It not only regulates innate and adaptive immune responses but also improves tolerance in immune reactions (7). Genetic evidence indicates a considerable causal interrelation between VD status and immune cells (8). Proteomics studies illustrated a negative interrelation between VD status and five immunoglobulins (JCHAJN, IGHV4-28, GHV4-34, IGHM, and IGLV2-11) (9). Nevertheless, the implication of VD for sepsis remains controversial. VD supplementation had no discernible implication on sepsis individuals’ mortality, as indicated by a recent network meta-analysis (10). However, previous meta-analyses have displayed a close interconnection between VD status and both the incidence and mortality of sepsis among both children and adults (11–13). Importantly, no studies have implemented a meta-analysis to estimate the forecasting capability of VD for the incidence and mortality of sepsis patients.

Therefore, our aim is to determine the interrelation between VD status and the risk of the occurrence and mortality of sepsis, and the predictive value of VD from observational studies, based on the guideline directions of early identification, rational intervention, and prognostic value, through systematic reviews and meta-analyses. This research calculates the pooled prevalence of VD deficiency and assesses VD status among sepsis individuals by combining means and standard deviations. This research also determines the relevance of supplementing VD on the length of ICU stay and mortality among sepsis individuals from randomized controlled trials (RCTs). Furthermore, considering age-related differences in sepsis patients, this study comprehensively assesses the relevance of VD in both pediatric and adult populations.

## 2 Methods

The design, implementation, and reporting of meta-analysis results in this research adhered to the Preferred Reporting Items for Systematic Reviews and Meta-Analyses (PRISMA) guidelines (14) (Appendix S1). This research was registered on the PROSPERO website^1^. The registration number was CRD420250651346.

### 2.1 Searching strategies

Related studies in PubMed, Embase, Cochrane Library, and Web of Science were searched by two researchers (AB), who were trained in systematic review approaches. The search terms were designed by combining medical subject headings (MeSH) terms and free terms. The search covered literature from database inception to February 2025. The search terms were as follows: VD OR vd OR 25-HydroxyVD AND sepsis OR Bloodstream Infection OR Septicemia. The specific search strategy is depicted in Supplementary Table S1. Reference lists from pertinent publications or reviews were manually searched as supplementary sources. Endnote 21 was leveraged to manage the retrieved references.

### 2.2 Inclusion and exclusion criteria

#### 2.2.1 Inclusion criteria

(i) The research subjects were individuals diagnosed with sepsis, regardless of age. The sepsis diagnosis must meet the criteria of SIRS, Sepsis-3, ICD-9, positive blood cultivation, or medical records indicating sepsis. The severity of sepsis consisted of severe sepsis and septic shock. In the control group, the “healthy cohort” was defined as individuals without any active or major chronic diseases, including known risk factors for sepsis. The “non-sepsis population” consisted of patients hospitalized for other reasons (e.g., elective surgery, stable chronic disease, or non-infectious acute conditions) but confirmed after assessment not to have sepsis.

(ii) The intervention or exposure factor involved VD supplementation or exposure to high levels of VD. VD deficiency was identified as a 25(OH)D level <20 ng/mL; insufficiency was identified as a 25(OH)D level of 20–30 ng/mL (15); severe deficiency was identified as a 25(OH)D level <12 ng/mL (16, 17); Due to the lack of established age specific vitamin D thresholds in sepsis patients of different age groups, the above uniform threshold values were applied to all included neonatal and pediatric populations.

(iii) The control group was not supplemented with VD or was exposed to inadequate levels of VD.

(iv) Outcome measures included VD levels in sepsis individuals, the prevalence of VD deficiency and insufficiency, the association and predictive value of VD with the incidence or mortality risk of sepsis, and the influence of VD supplementation on length of ICU stay and mortality in sepsis patients.

(v) Study types encompassed cohort studies, cross-sectional studies, case–control studies, and RCTs.

#### 2.2.2 Exclusion criteria

(i) Review articles, clinical registration protocols, guidelines, and other types of studies.

(ii) Studies lacking complete data on odds ratios (OR), relative risks (RR), hazard ratios (HR), or their 95% confidence intervals.

(iii) Duplicated publications.

### 2.3 Literature screening

Two researchers (A and B) separately screened the retrieved literature records utilizing pre-established inclusion and exclusion criteria. If dissents occurred, a third researcher (C) was engaged to resolve the issue. EndNote 21 was utilized as the reference management tool for this study, inputting the initial search results. Both researchers independently searched for duplicate records, screened the titles and abstracts of the retrieved articles, and further appraised eligible studies by downloading and reading the complete text.

### 2.4 Data collection process

Two researchers (A and B) independently carried out the data extraction process and subsequently organized and collated the extracted data. The subsequent data information was obtained from studies that complied with the inclusion criteria: first author, publication year, study type, country/region, age, sample size, sepsis type, diagnostic criteria for sepsis, type of VD, and outcome measures [(i) Mean and standard deviation were extracted to assess VD status and length of ICU stay among sepsis individuals; (ii) The number of events and total number of participants were extracted to appraise the prevalence of VD deficiency; (iii) Binary variables were extracted to estimate the interrelation between VD and mortality in sepsis patients; (iv) Both multivariable-adjusted and unadjusted results were extracted to appraise the link between VD and the incidence and mortality of sepsis; (v) For diagnostic tests, true positives, false negatives, true negatives, false positives, sensitivity, and specificity were extracted to estimate the predictive value of VD for the incidence and mortality of sepsis]. Any disputes were addressed by discussing with a third investigator (C).

### 2.5 Quality appraisal

Two researchers (A and B) separately estimated the quality and methodological rigor of the included articles. The Newcastle–Ottawa scale (NOS) was leveraged to appraise the quality of cohort studies and case–control studies (18). The scale encompassed three domains: the selection of research groups, the comparability of the exposed (case) group and the control group achieved by adjusting for confounding factors, and the ascertainment of outcomes of interest. Articles with a score of 7 or more were rated as high quality. A score of 4–6 denoted moderate quality, and 0–3 signified low quality. Cross-sectional studies were appraised by leveraging the Agency for Healthcare Research and Quality (AHRQ) assessment tool (19). This tool contained 11 items. A score of 8–11 denoted high quality, 4–7 indicated moderate quality, and 0–3 denoted low quality. For RCT studies, the Cochrane risk of bias tool (RoB2.0) was leveraged (20). This tool assessed the risk of bias (RoB) in five domains: (i) bias originating from randomization process; (ii) bias originating from deviations from the intended interventions; (iii) bias originating from missing outcome data; (iv) bias in outcomes measurement; (v) bias in the reporting of results. In case of any disagreements, a third investigator (C) would be consulted to make the final decision.

### 2.6 Statistical analyses

Statistical analysis was implemented utilizing Stata 15 and Meta-Disc 1.4 software. The *I*^2^ statistic and Cochran *Q* test were leveraged to estimate the heterogeneity of the included studies. *I*^2^ of 25–50% denoted low heterogeneity; *I*^2^ of 50–75% signified moderate heterogeneity, and *I*^2^ > 75% denoted high heterogeneity (21). *p* < 0.05 or *I*^2^ > 50% signified significant heterogeneity between studies, and a random-effects model was leveraged. Otherwise, a fixed-effects model was applied. Sensitivity analysis was implemented utilizing the leave-one-out method for outcomes with more than five included studies, in order to ascertain the stability of the results (22). Additionally, subgroup analysis was conducted by the degree of VD deficiency and control groups (healthy individuals, non-sepsis groups) to estimate the relevance of these characteristics on the outcomes and whether they were sources of heterogeneity. For meta-analyses including over 10 studies, potential publication bias was appraised. The Egger’s test was leveraged to further estimate publication bias (23). For diagnostic tests, Spearman correlation analysis was implemented utilizing Meta-Disc 1.4 software to assess threshold effects, where a strong positive correlation indicated the possibility of a threshold effect. If no threshold effect was found, data were combined for further analysis. A bivariate mixed-effects model was leveraged to summarize the effect sizes, including the summary sensitivity (SSEN) and summary specificity (SSPE). Deeks’ funnel plot was utilized to examine publication bias.

## 3 Results

### 3.1 Literature screening

This study initially retrieved 2,890 publications, of which 872 were from PubMed, 1724 from Embase, 188 from Cochrane, and 106 from Web of Science. After excluding duplicates (*n* = 369) and removing others for various reasons (*n* = 153), we reviewed the titles and abstracts of 2,368 publications. After excluding 2,133 studies, 235 articles were reviewed fully. Twenty-one articles were eliminated owing to the unavailability of their whole texts, and we examined 214 publications in detail. Finally, 39 eligible articles were incorporated into this analysis (Supplementary Figure S1).

### 3.2 Research characteristics

This study divided the baseline information into two parts based on age characteristics: neonates and children, and adults.

Among neonates and children, 10 studies were included, consisting of five cohort studies (24–28), four case–control studies (29–32), and one cross-sectional study (33). Six studies were carried out in India (24, 27, 28, 30–32), two in Turkey (25, 26), one in Egypt (29), and one in Iran (33). Eight studies reported levels of VD (25, 26, 28–33). Seven studies reported the prevalence of VD deficiency (24, 26–28, 30–32). Four studies reported the risk of mortality due to VD deficiency (24, 27, 28, 32).

In adult sepsis patients, 29 studies were included, comprising 24 cohort studies (34–57), three cross-sectional studies (58–60), one case–control study (61), and one RCT (62). Seven studies were carried out in the United States (34–37, 39, 48, 53), three in China (41, 55, 56), three in Thailand (46, 59, 60), three in India (52, 61, 62), two in Brazil (38, 40), two in Turkey (49, 54), two in South Korea (50, 57), one in the Netherlands (42), one in Romania (43), one in Germany (44), one in Australia (45), one in Bahrain (47), one in Iran (58), and one in Italy (51). Eighteen studies reported levels of VD (34, 36, 39–41, 44–47, 49, 50, 52, 54, 57–61). Seventeen studies reported the prevalence of VD deficiency and insufficiency (34, 36, 38–43, 46–48, 52, 56–58, 60, 61). Two studies reported the VD deficiency and the risk of sepsis onset through multivariate statistical analysis (35, 39). Twelve studies reported VD and the mortality risk of sepsis based on binary variables (37, 41, 44, 46, 47, 51, 52, 57). Four studies reported the VD supplementation and the mortality risk of sepsis (53, 55, 56, 62). Eight studies reported the VD status and the mortality risk of sepsis through multivariate statistical analysis (35, 41–43, 52, 54, 57, 58). Four studies investigated the relevance of VD supplementation on the length of ICU stay in individuals with sepsis (53, 55, 56, 62). Three studies reported the forecasting capability of VD for mortality in individuals with sepsis (37, 58) (Table 1).

**Table 1:** Basic characteristics of studies included.

Age subgroups	First author	Publication year	Study Type	Country/region	Age (range, mean ± SD) at baseline	Sample size	Participants for analysis	Diagnosis of sepsis	Vitamin D type	Outcome
Neonatal and pediatric sepsis patients	Satheesh	2013	Cohort study	India	5.30 ± 5.53y	164	Sepsis	Medical record	25-OH-D	②④
	Aydemir	2014	Cohort study	Turkey	6.67 ± 2.66y	60	Sepsis	Blood Culture	25-OH-D	①
	Gamal	2017	Case control study	Egypt	37.46 ± 0.85w	80	Neonatal sepsis	Medical record	25-OH-D	①
	Prasad	2018	Case control study	India	34–36w	120	Neonatal sepsis	Blood Culture	25-OH-D	①②
	Agrawal	2019	Case control study	India	8.89 ± 4.83d	175	Neonatal sepsis	Blood Culture	25-OH-D	①②
	Ozdemir	2019	Cohort study	Turkey	38.15 ± 1.2w	107	Neonatal sepsis	Medical record	25-OH-D	①②
	Kumar	2020	Cohort study	India	≤12y	195	Sepsis	Medical record	25-OH-D	②④
	Singh	2020	Cohort study	India	NA	70	Neonatal sepsis	SIRS	25-OH-D	①②④
	Kubsad	2021	Case control study	India	4.32 ± 5y	168	Sepsis	SOFA	25-OH-D	①②④
	Zakerihamidi	2023	Cross-sectional	Iran	≤34w	69	Neonatal sepsis	Blood Culture	25-OH-D	①
Adult sepsis	Kempker	2012	Cohort study	America	≥18y	41	Sepsis	Medical record	25-OH-D	①②
	Christopher	2012	Cohort study	America	≥18y	2,399	Sepsis	Medical record	25-OH-D	③⑥
	Salciccioli	2012	Cohort study	America	67.64 ± 17.7y	39	Sepsis	SIRS	25-OH-D	①②
	Nguyen	2013	Cohort study	America	59.10 ± 2.0y	91	Sepsis	SIRS	25-OH-D	④⑧
	Alves	2013	Cohort study	Brazil	51y	34	Sepsis	Medical record	25-OH-D	②
	Jovanovich	2014	Cohort study	America	65 ± 14y	422	Sepsis	ICD-9	25-OH-D	①②③
	Alves	2015	Cohort study	Brazil	43.96 ± 32.41y	51	Sepsis	ACCP/SCCM	25-OH-D	①②
	Chen	2015	Cohort study	China	62.38 ± 13.06y	236	Sepsis	the Surviving Sepsis Campaign	25-OH-D	①②④⑥
	De Haan	2015	Cohort study	Holland	≥18y	940	Sepsis	Medical record	25-OH-D	②⑥
	Pascale	2016	Cohort study	Rome	66.69 ± 13.08y	107	Sepsis	Medical record	25-OH-D	②⑥
	Greulich	2017	Cohort study	Germany	61.26 ± 13.86y	64	Sepsis	ACCP/SCCM	25-OH-D	①④
	Ratzinger	2017	Cohort study	Austria	56.45 ± 19.39y	461	Sepsis	SIRS	25-OH-D	①
	Trongtrakul	2017	Cohort study	Thailand	58.49 ± 15.04y	114	Sepsis	the Surviving Sepsis Campaign	25-OH-D	①②④⑥
	Mohapatra	2018	Case control study	India	51.76 ± 18.87y	119	Sepsis; Severe sepsis; Septic Shock	ACCP/SCCM	25-OH-D	①②
	Anis	2018	Cohort study	Bahrain	69.47 ± 12.79y	33	Sepsis	Medical record	25-OH-D	①②④
	Pinargote	2018	Cohort study	America	64.4y	10,814	Sepsis	Medical record	25-OH-D	②
	GUL	2019	Cohort study	Turkey	55.74 ± 17.81y	45	Sepsis	the 2001 International Sepsis Definitions	25-OH-D	①
	Shojaei	2019	Cross-sectional	Iran	70.8 ± 13.3y	168	Sepsis	Blood Culture	25-OH-D	①②⑥⑧
	Yoo	2020	Cohort study	Korea	70.97 ± 15.20y	98	Sepsis	Blood Culture	25-OH-D	①
	Romposra	2020	Cross-sectional	Thailand	67.9 ± 18.20y	101	Sepsis	Medical record	25-OH-D	①
	Bhattacharyya	2021	RCT	India	43.16 ± 18.21y	126	Sepsis	Medical record	VD3	⑤⑦
	Tosoni	2021	Cohort study	Italy	75y	80	Sepsis	SIRS	25-OH-D	④⑧
	Asdie	2023	Cohort study	India	56.09 ± 16.82y	88	Sepsis	Sepsis-3	25-OH-D	①②④⑥
	Guan	2023	Cohort study	America	62.07 ± 13.58y	19,816	Sepsis	Sepsis-3	VD3	⑤⑦
	Kahar	2023	Cohort study	Turkey	50.89 ± 12.54y	80	Sepsis	Medical record	25-OH-D	①⑥
	Vanichkulbodee	2023	Cross-sectional	Thailand	68 ± 18y	101	Sepsis	SIRS	25-OH-D	①②
	Seok	2023	Cohort study	Korea	74 ± 13y	129	Sepsis	Sepsis-3、SOFA	25-OH-D	①②④⑥
	Yang	2023	Cohort study	China	68 ± 17.80y	3,539	Sepsis	Medical record	25-OH-D	⑤⑦
	Li	2025	Cohort study	China	≥18y	20,230	Sepsis	Sepsis-3	25-OH-D	②⑤⑦

RCT, randomized controlled trial; SIRS, systemic inflammatory response syndrome; SOFA, sequential organ failure assessment; ICD-9, international classification of diseases, ninth revision; ACCP/SCCM, the American College of Chest Physicians and the Society of Critical Care Medicine.

①: Vitamin D levels; ②: Vitamin D deficiency and insufficient rate; ③: Vitamin D deficiency and the Risk of Sepsis Incidence on Multivariate Analysis; ④: Based on the binary variable of vitamin D levels and sepsis mortality; ⑤: Supplementing Vitamin D with Binary Variables and Sepsis related Death; ⑥: Vitamin D and the Risk of Mortality from Sepsis Based on Multivariate Analysis; ⑦: ICU length of stay; ⑧: The Predictive Value of Vitamin D for Sepsis Mortality.

### 3.3 RoB appraisal of included studies

The RoB of cohort studies and case–control studies was appraised utilizing the NOS scale (Supplementary Tables S2, S3). Among the 29 cohort studies, 20 were of high quality and nine were of moderate quality. Among the five case–control studies, one was of high quality and four were of moderate quality. The four cross-sectional studies were assessed for RoB utilizing the AHRQ tool, among which two studies were of high quality and two were of moderate quality (Supplementary Table S4). A RoB assessment of one RCT study was conducted utilizing the ROB2 tool. The RCT was assessed to have a low RoB in domains such as bias originating from the randomization process, bias originating from deviations from the intended interventions, bias originating from missing outcome data, bias in outcome measurement, and bias in the reporting of outcomes (Supplementary Figure S2).

### 3.4 Neonatal and pediatric sepsis patients

#### 3.4.1 VD levels

**Figure 1:**
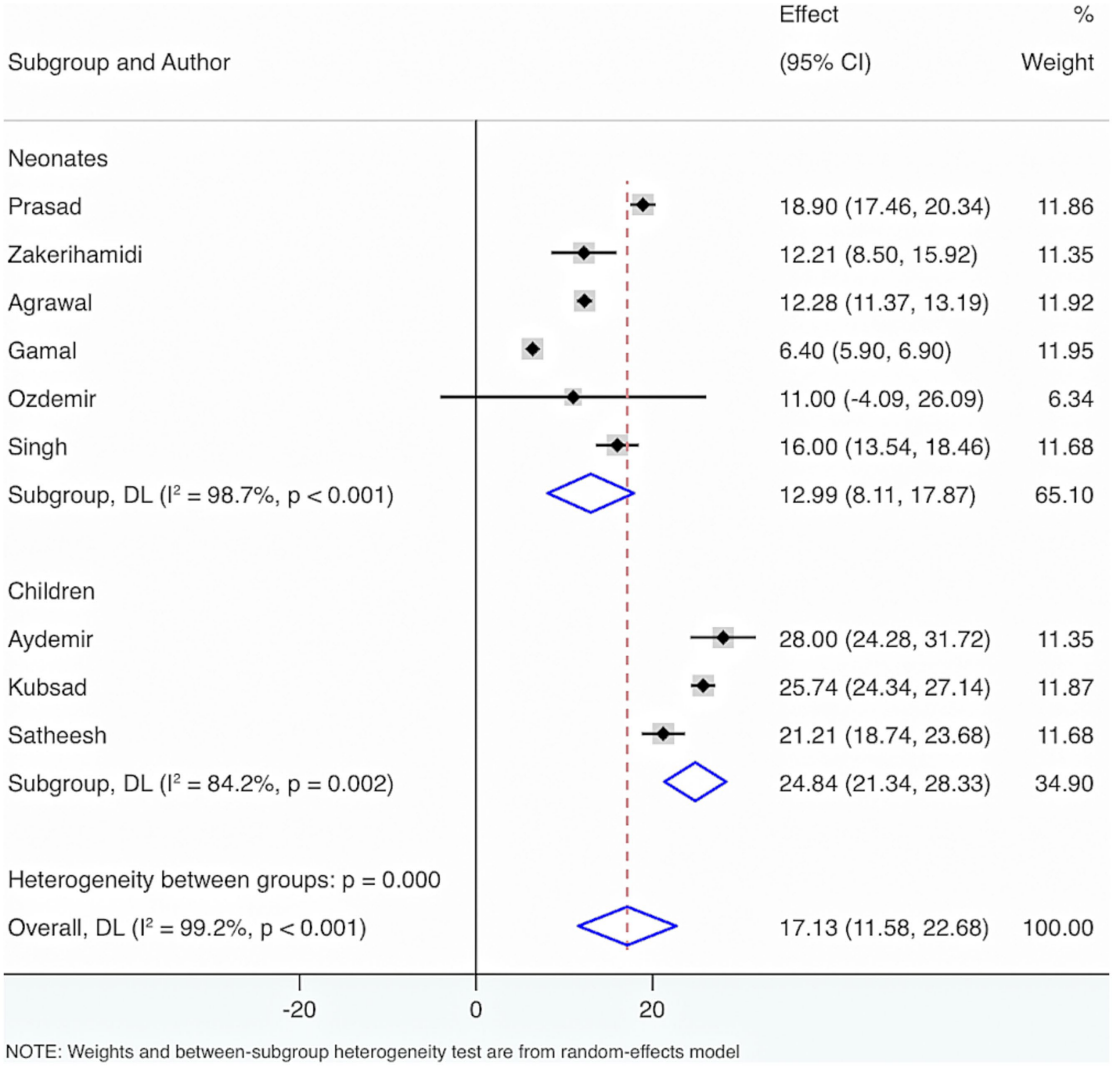
The vitamin D (VD) status among neonates and children having sepsis.

Nine studies were included to estimate the VD status among neonates and children having sepsis. The outcomes of the Cochrane Q test (*p* < 0.001) and the *I*^2^ estimate (99.2%) indicated significant heterogeneity. The random-effects model was leveraged for meta-analysis. The results showed that the average VD level in neonates with sepsis was 12.99 (95% CI: 8.11, 17.87), and the average VD level in children was 24.84 (95% CI: 21.34, 28.33) (Figure 1).

Further subgroup analyses were made by control groups comprising healthy individuals or neonates and children without sepsis, based on seven included studies. The Cochrane *Q* test (*p* < 0.001) and *I*^2^ estimate (97.5%) also displayed considerable heterogeneity. The random-effects meta-analysis indicated that neonates and children suffering from sepsis had considerably diminished VD levels relative to control groups, with a statistical distinction (SMD = −2.29, 95% CI: −3.31, −1.28, *p* < 0.001). Subgroup analysis revealed that, compared with healthy individuals, the sepsis group had a considerably lower VD level with statistical distinction (SMD = −3.16, 95% CI: −5.33, −1.00, *p* = 0.004). Additionally, when compared with those without sepsis, the sepsis group also exhibited significantly lower VD levels with a statistical distinction (SMD = −1.83, 95% CI: −3.06, −0.61, *p* = 0.003).

**Figure 2:**
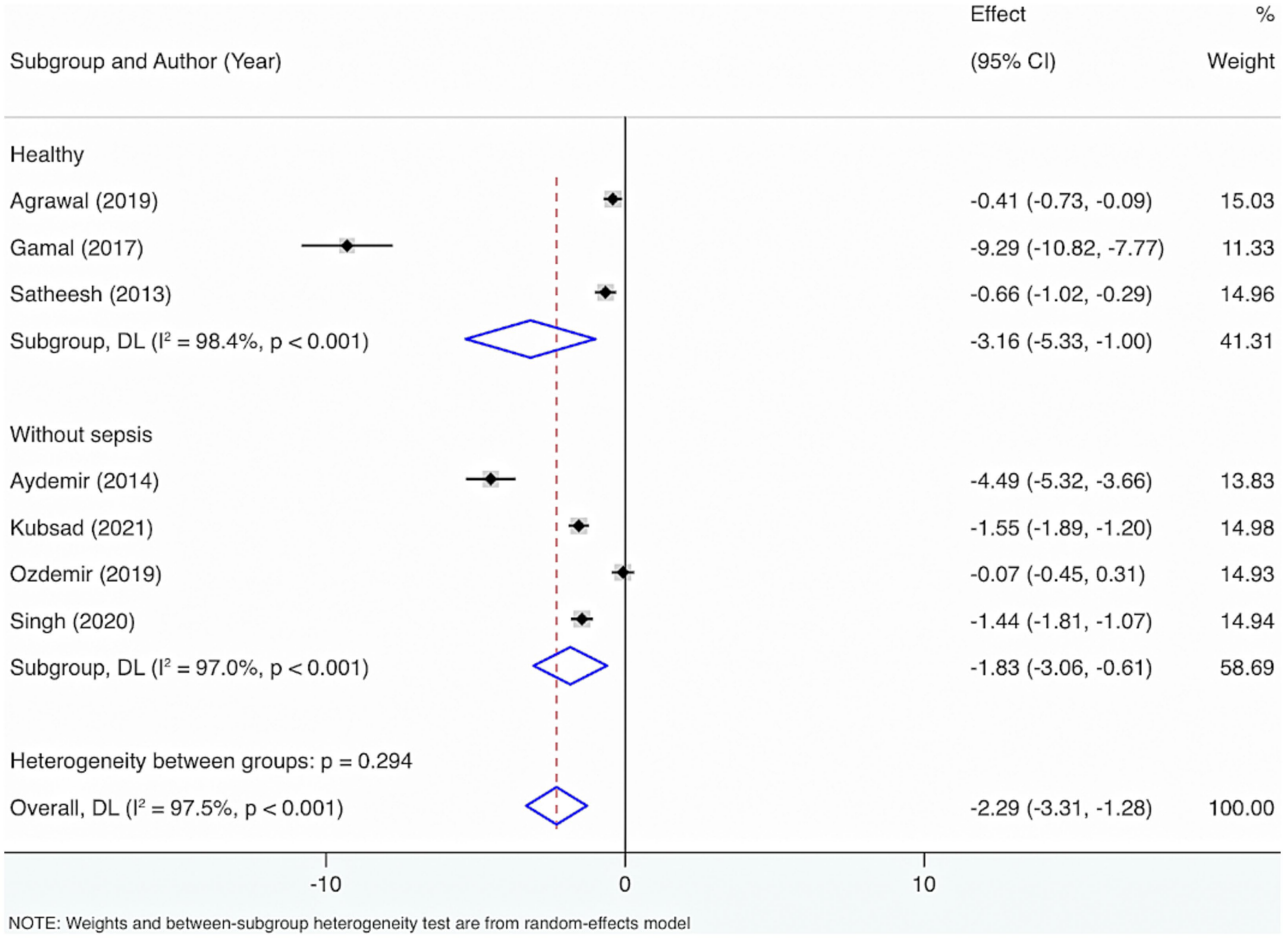
Subgroup analyses were made by control groups comprising healthy individuals or neonates and children without sepsis.

Furthermore, to figure out the source of heterogeneity, when the control group consisted of healthy individuals, *I*^2^ = 98.4%, *p* < 0.001; and when the control group consisted of individuals without sepsis, *I*^2^ = 97%, *p* < 0.001. This suggests that the control group may not be the primary source of the high heterogeneity (Figure 2).

#### 3.4.2 Prevalence of VD deficiency and insufficiency

**Figure 3:**
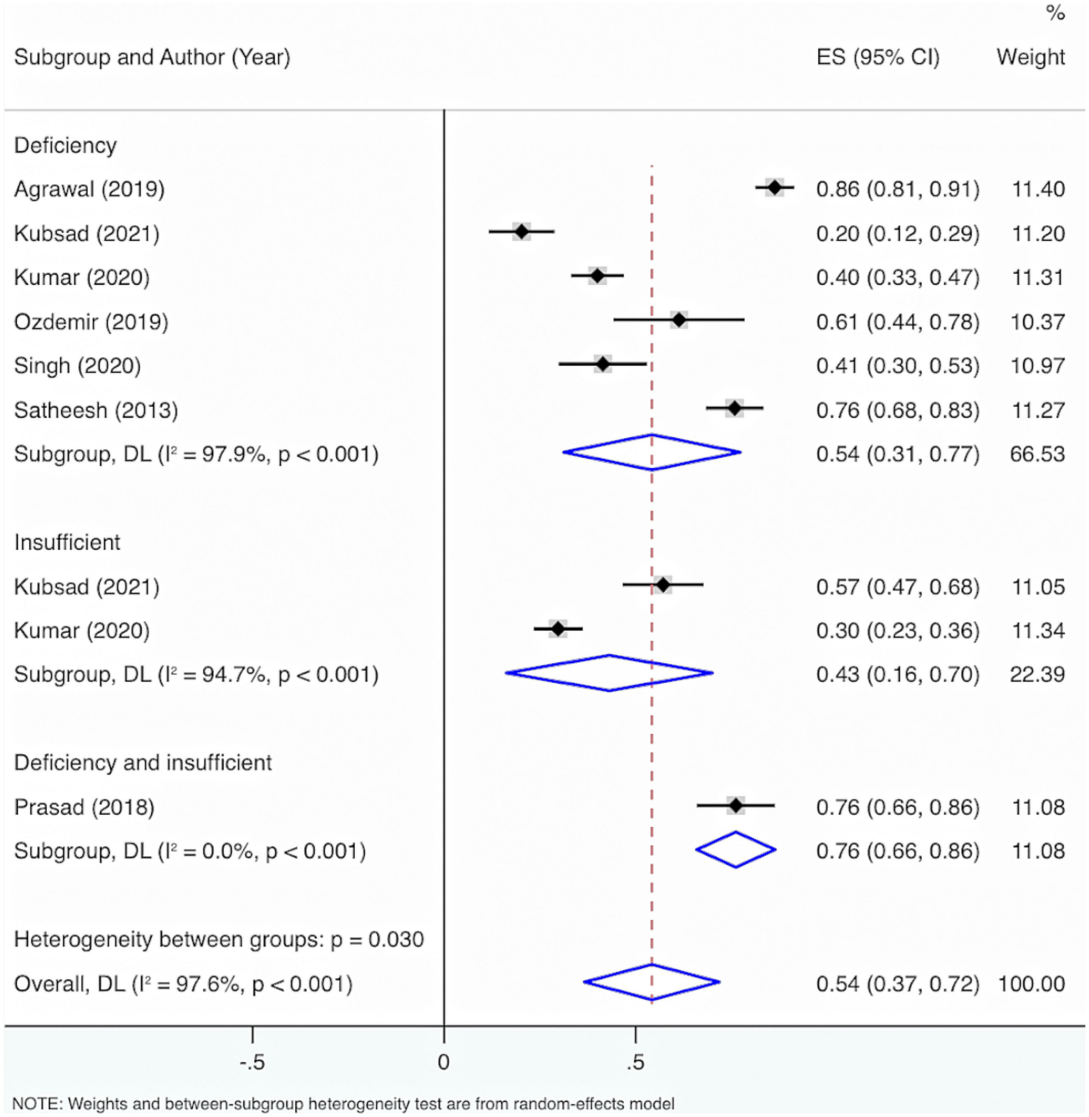
Prevalence of VD deficiency and insufficiency among neonates and children having sepsis.

Seven studies were included. The results from the Cochrane *Q* test (*p* < 0.001) and *I*^2^ estimate (97.6%) signified considerable heterogeneity. Meta-analysis utilizing a random-effects model displayed that the overall prevalence of VD deficiency and insufficiency among neonatal and pediatric sepsis patients was 54% (95% CI: 37, 72%, *p* < 0.001) (Figure 3).

Subgroup analysis grounded in the characteristics of deficiency and insufficiency displayed that the aggregated prevalence of VD deficiency was 54% (95% CI: 31, 77%, *p* < 0.001). The overall prevalence of insufficiency was 43% (95% CI: 16, 70%, *p* = 0.002). The overall prevalence of either deficiency or insufficiency was 76% (95% CI: 66, 86%, *p* < 0.001).

**Figure 4:**
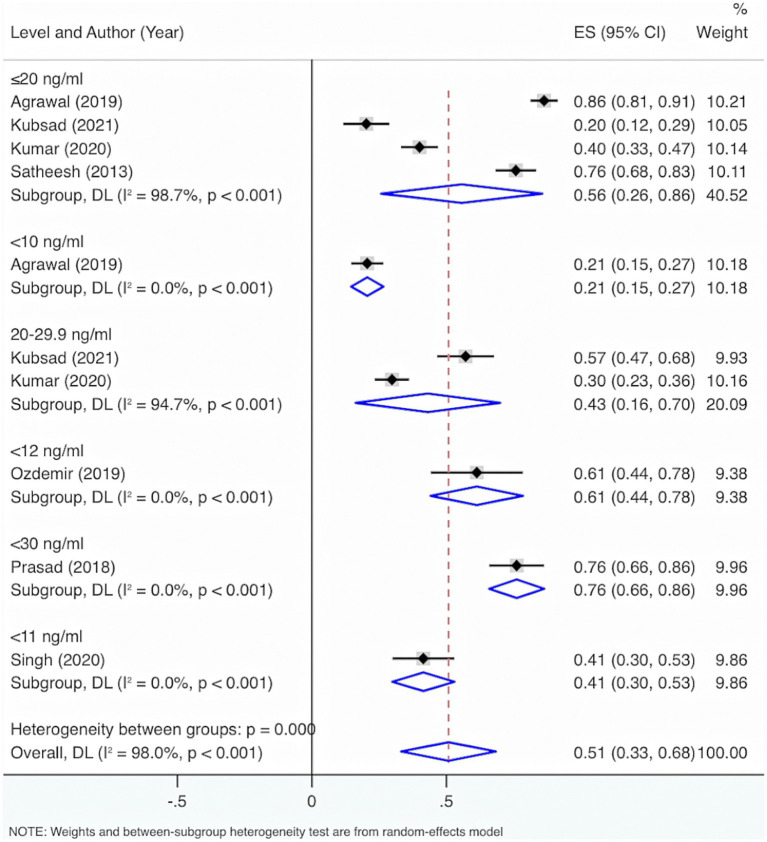
Subgroup analysis grounded in the characteristics of deficiency and insufficiency.

Subgroup analysis grounded in VD levels displayed that when VD levels were <30 ng/mL, <12 ng/mL, or ≤20 ng/mL, the overall prevalence of deficiency and insufficiency was relatively high, at 76% (95% CI: 66, 86%, *p* < 0.001), 61% (95% CI: 44, 78%, *p* < 0.001), and 54% (95% CI: 26, 86%, *p* < 0.001), respectively (Figure 4).

Further exploration of heterogeneity sources showed that in the features of deficiency and insufficiency, the heterogeneity result for deficiency was *I*^2^ = 97.9%, *p* < 0.001, for insufficiency was *I*^2^ = 94.7%, *p* < 0.001, and for deficiency or insufficiency was *I*^2^ = 0%, *p* < 0.001. Consequently, this may not be the principal cause of the high heterogeneity. The heterogeneity results were *I*^2^ = 0%, *p* < 0.001 for VD levels <30 ng/mL; *I*^2^ = 94.7%, *p* < 0.001 for VD levels of 20–29.9 ng/mL; *I*^2^ = 98.7%, *p* < 0.001 for VD levels ≤20 ng/mL; *I*^2^ = 0%, *p* < 0.001 for VD levels <12 ng/mL; *I*^2^ = 0%, *p* < 0.001 for VD levels <11 ng/mL; *I*^2^ = 0%, *p* < 0.001 for VD levels <10 ng/mL. Therefore, VD level was also not the main reason for the high heterogeneity.

#### 3.4.3 VD deficiency and risk of mortality

**Figure 5:**
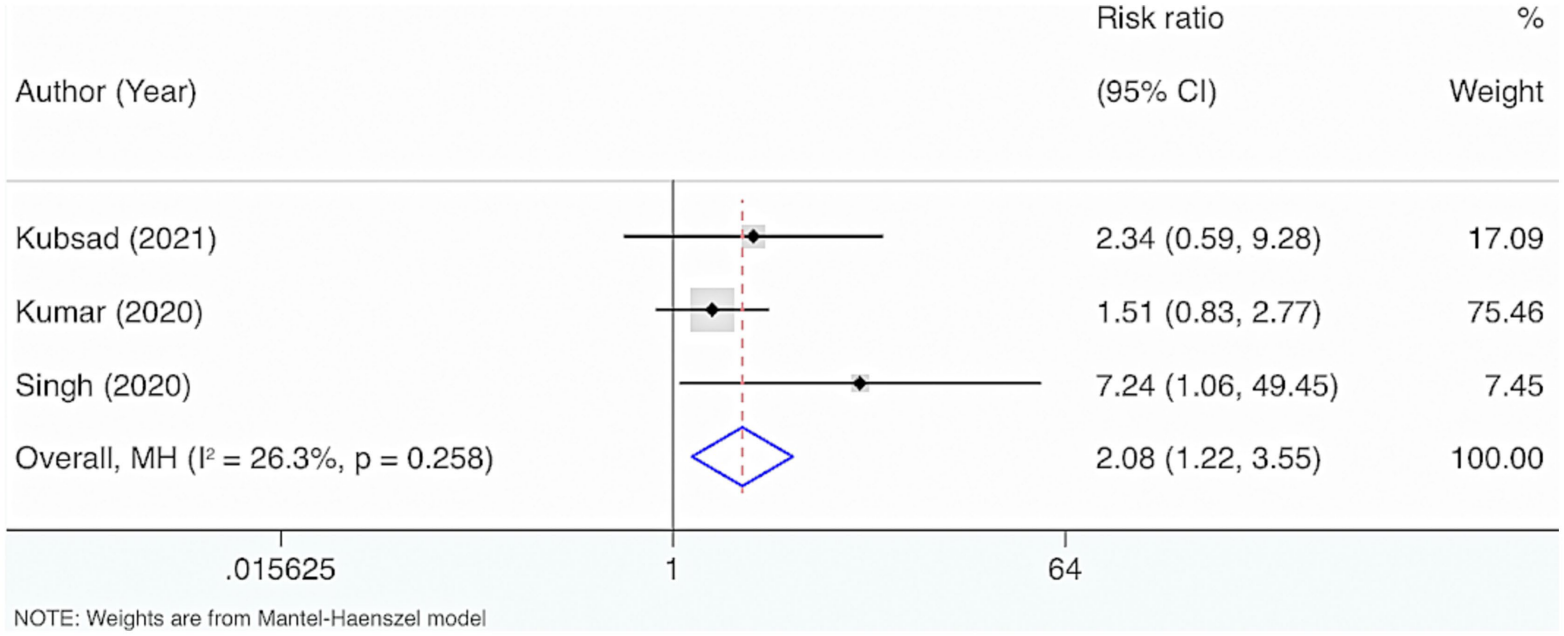
Vitamin D deficiency and risk of mortality among neonates and children having sepsis.

Three studies were included. The results from the Cochrane *Q* test (*p* = 0.26) and I^2^ estimate (26.3%) displayed no significant heterogeneity. Meta-analysis leveraging a fixed-effects model indicated a marked link between the deficiency of VD and mortality in neonatal and pediatric sepsis patients compared to the control group (RR = 2.08, 95% CI: 1.22, 3.55, *p* = 0.007) (Figure 5).

### 3.5 Adult sepsis

#### 3.5.1 VD levels

**Figure 6:**
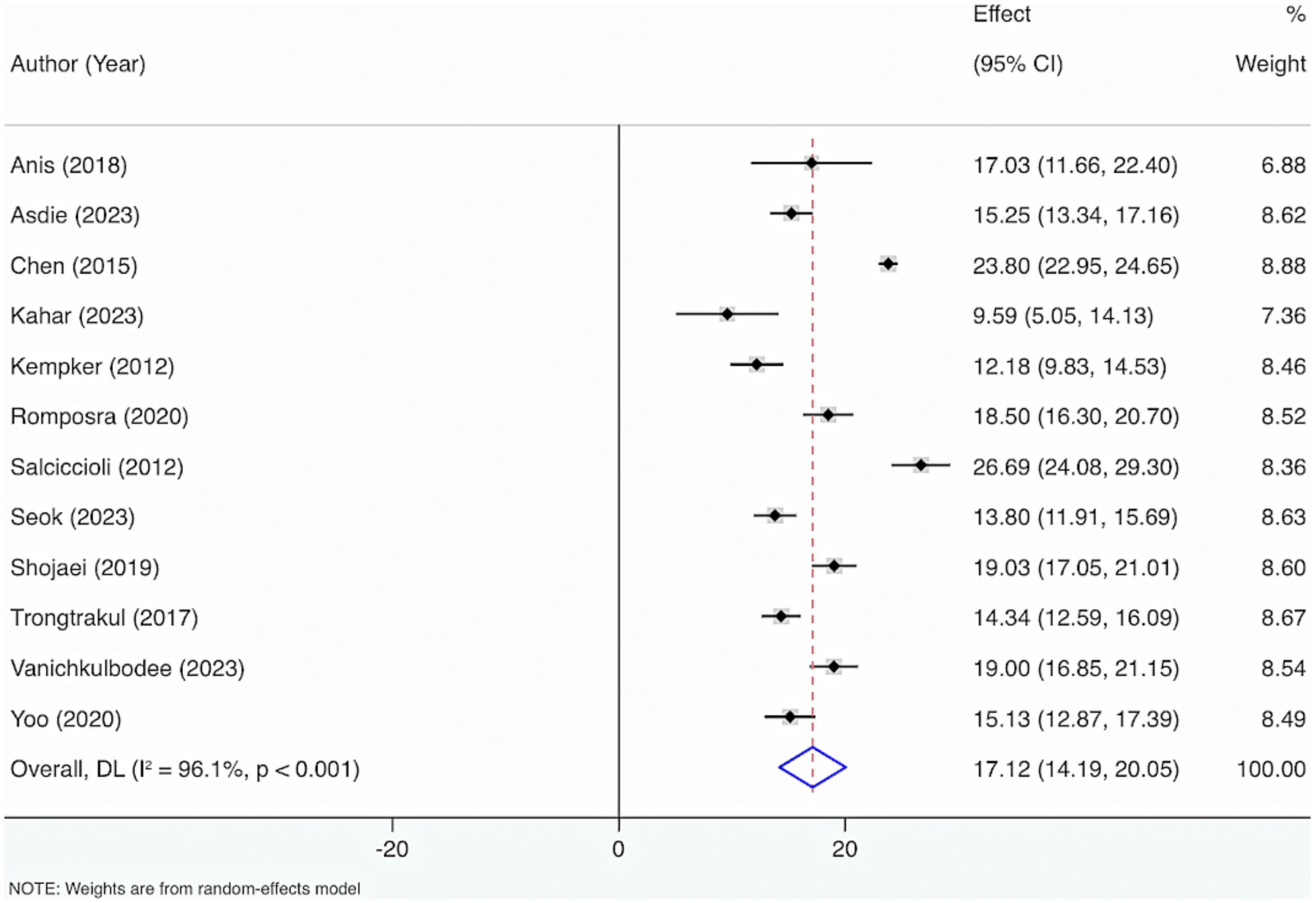
Vitamin D levels in adult sepsis individuals.

Twelve studies were included to estimate VD levels in adult sepsis individuals. The results from the Cochrane Q test (*p* < 0.001) and I^2^ estimate (96.1%) displayed considerable heterogeneity. Meta-analysis leveraging a random-effects model displayed that the average level of VD was 17.12 (95% CI: 14.19, 20.05) (Figure 6).

Seven studies were included to perform subgroup analysis by the control group, encompassing healthy individuals and those without sepsis. The results from the Cochrane *Q* test (*p* < 0.001) and *I*^2^ estimate (96.0%) showed considerable heterogeneity. Meta-analysis leveraging a random-effects model displayed that VD levels were considerably diminished in sepsis individuals relative to healthy individuals or those without sepsis (SMD = −1.63, 95% CI: −2.39, −0.87, *p* < 0.001), with considerable statistical distinction. Subgroup analysis showed that compared to healthy individuals, VD levels were considerably lower in adults with sepsis, with statistical differences (SMD = −3.32, 95% CI: −6.44, −0.19, *p* = 0.04); relative to people without sepsis, there was no statistically distinction in VD levels (SMD = −0.77, 95% CI: −1.55, 0.01, *p* = 0.05).

**Figure 7:**
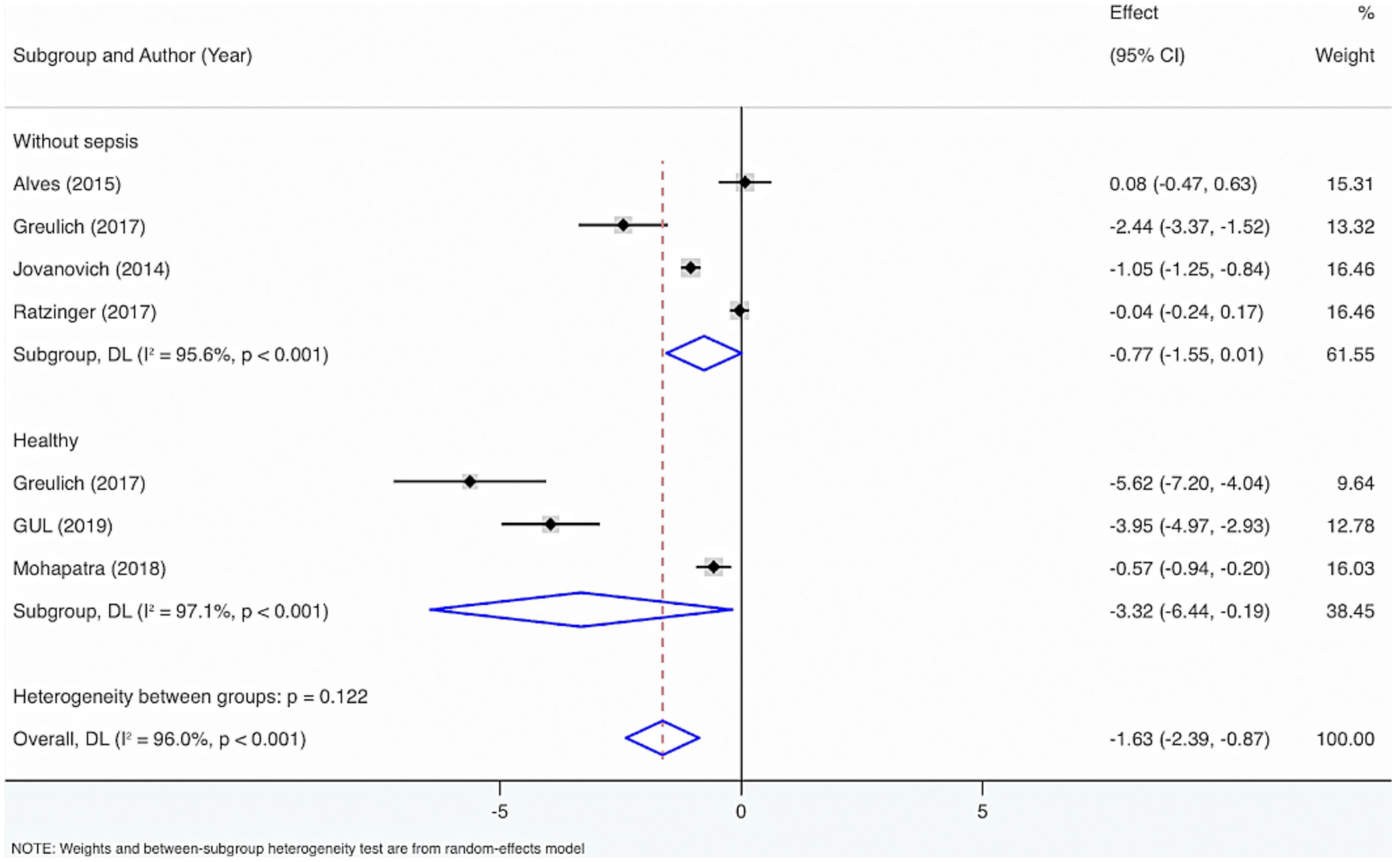
Subgroup analysis of control group consisting of healthy adults or adults without sepsis.

In addition, to figure out the source of heterogeneity, when the control group comprised healthy individuals, *I*^2^ = 97.1%, *p* < 0.001. When the control group comprised individuals without sepsis, *I*^2^ = 95.6%, *p* < 0.001. Therefore, the control group may not cause the high heterogeneity (Figure 7).

#### 3.5.2 Prevalence of VD deficiency and insufficiency

Seventeen studies were included. The results from the Cochrane *Q* test (*p* < 0.001) and *I*^2^ estimate (99.9%) showed significant heterogeneity. Meta-analysis was executed by leveraging a random-effects model. The results unraveled an overall prevalence of VD deficiency and insufficiency of 55% (95% CI: 39, 71%, *p* < 0.001).

Subgroup analysis by characteristics of deficiency and insufficiency displayed that the overall prevalence of VD deficiency was 52% (95% CI: 34, 71%, *p* < 0.001). The overall prevalence of insufficiency was 43% (95% CI: 24, 63%, *p* < 0.001). The overall prevalence of either deficiency or insufficiency was 80% (95% CI: 67, 93%, *p* < 0.001).

**Figure 8:**
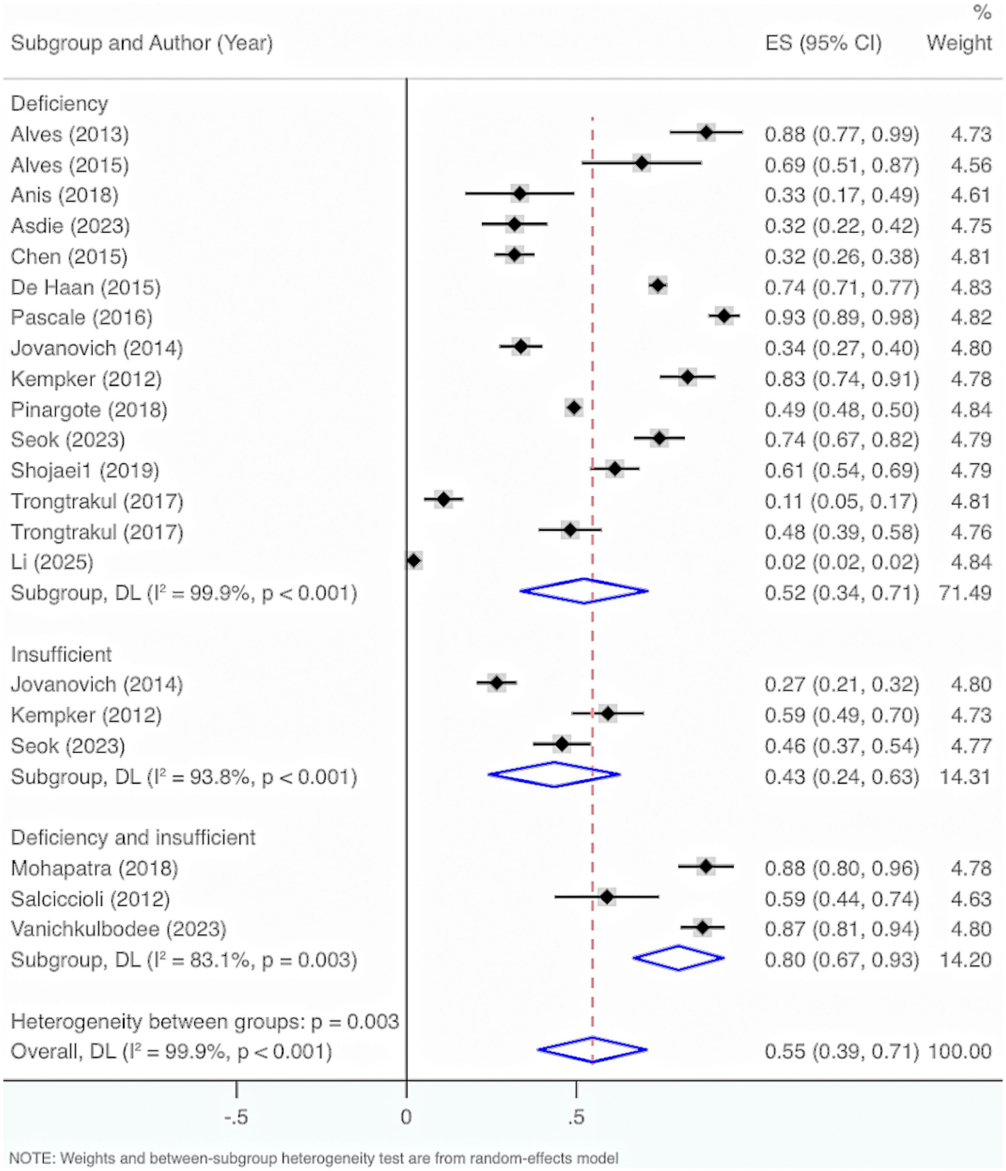
Prevalence of VD deficiency and insufficiency among adult sepsis individuals.

Further exploration of heterogeneity sources illustrated that in the features of deficiency and insufficiency, the heterogeneity result for deficiency was *I*^2^ = 99.9%, *p* < 0.001, for insufficiency was *I*^2^ = 93.8%, p < 0.001, and for deficiency or insufficiency was *I*^2^ = 83.1%, *p* < 0.001. Therefore, this may not be the main cause of the high heterogeneity (Figure 8).

#### 3.5.3 VD deficiency and incidence of sepsis

**Figure 9:**
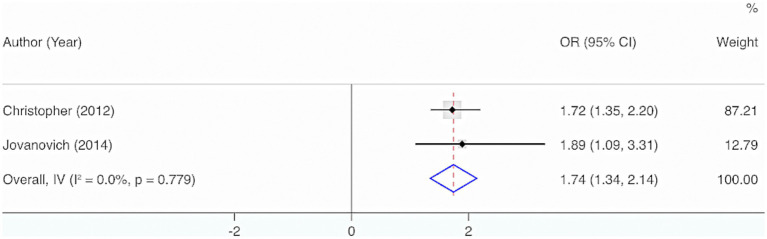
Vitamin D deficiency and incidence rate of adult sepsis.

Two studies were included. The results from the Cochrane *Q* test (*p* = 0.78) and *I*^2^ estimate (0%) displayed no significant heterogeneity. Meta-analysis utilizing a fixed-effects model indicated a considerable interrelation between the deficiency of VD and the incidence of sepsis in adults (OR = 1.74, 95% CI: 1.34, 2.14, *p* < 0.001) (Figure 9).

#### 3.5.4 VD levels and sepsis mortality

**Figure 10:**
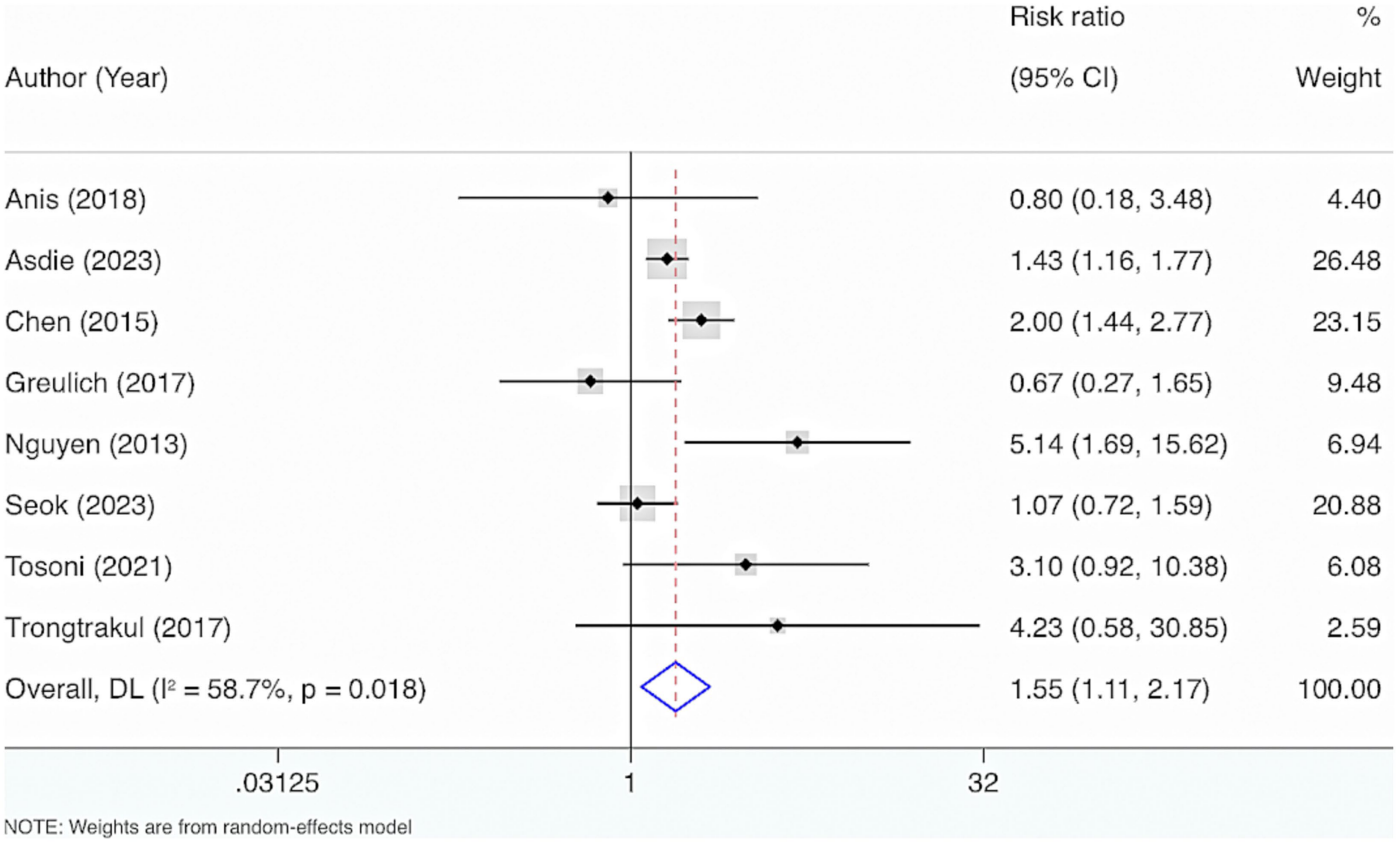
The relationship between VD levels and adult sepsis mortality.

Eight studies were included. The results from the Cochrane *Q* test (*p* = 0.02) and *I*^2^ estimate (58.7%) displayed considerable heterogeneity. Meta-analysis leveraging a random-effects model displayed a considerable interrelation between reduced VD levels and mortality among adult individuals with sepsis (RR = 1.55, 95% CI: 1.11, 2.17, *p* = 0.01) (Figure 10).

#### 3.5.5 VD supplementation and mortality of sepsis

**Figure 11:**
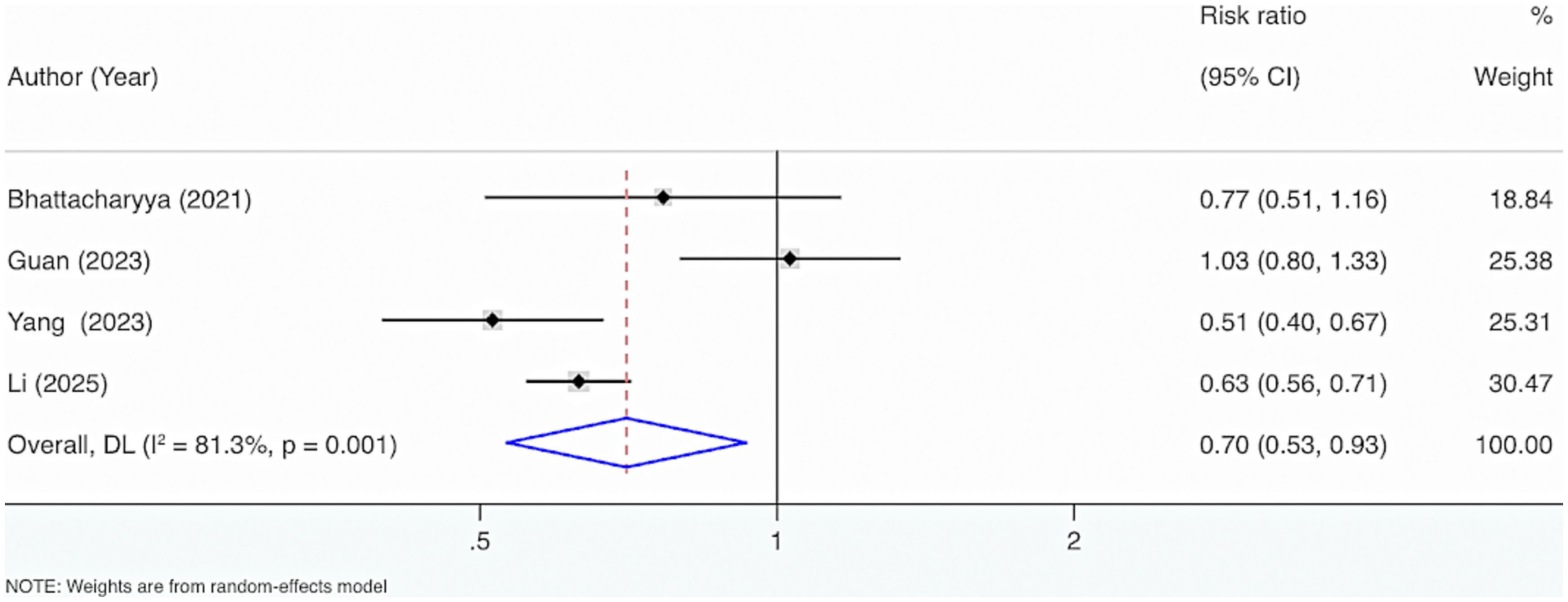
The relationship between VD supplementation and adult sepsis mortality.

Four studies were included. The results from the Cochrane *Q* test (*p* = 0.001) and I^2^ estimate (81.3%) showed considerable heterogeneity. Meta-analysis leveraging a random-effects model displayed that VD supplementation considerably reduced the occurrence of mortality events (RR = 0.70, 95% CI: 0.53, 0.93, *p* = 0.01) (Figure 11).

#### 3.5.6 Deficiency of VD and mortality of sepsis

**Figure 12:**
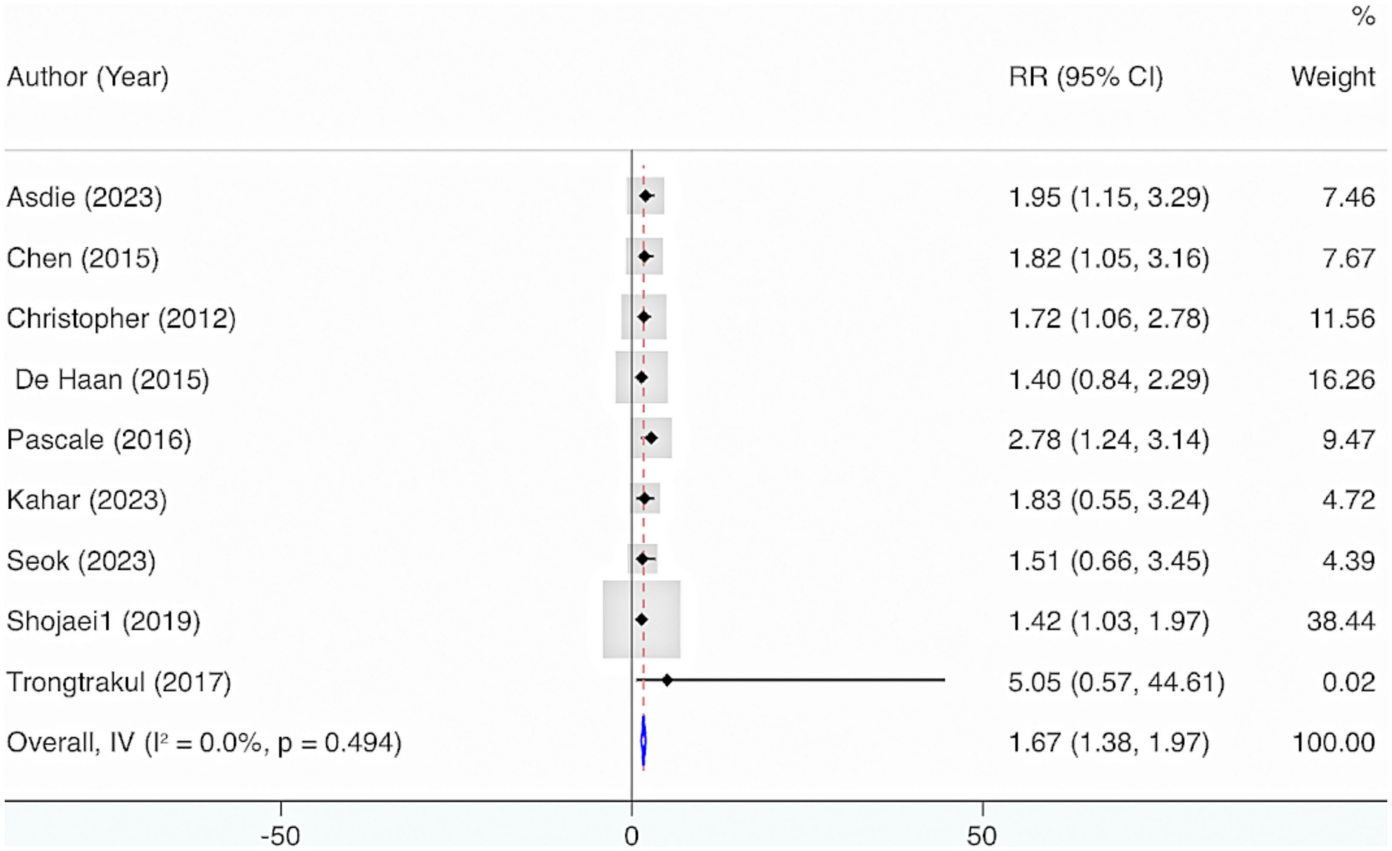
The relationship between VD deficiency and the risk of mortality in adult sepsis.

**Figure 13:**
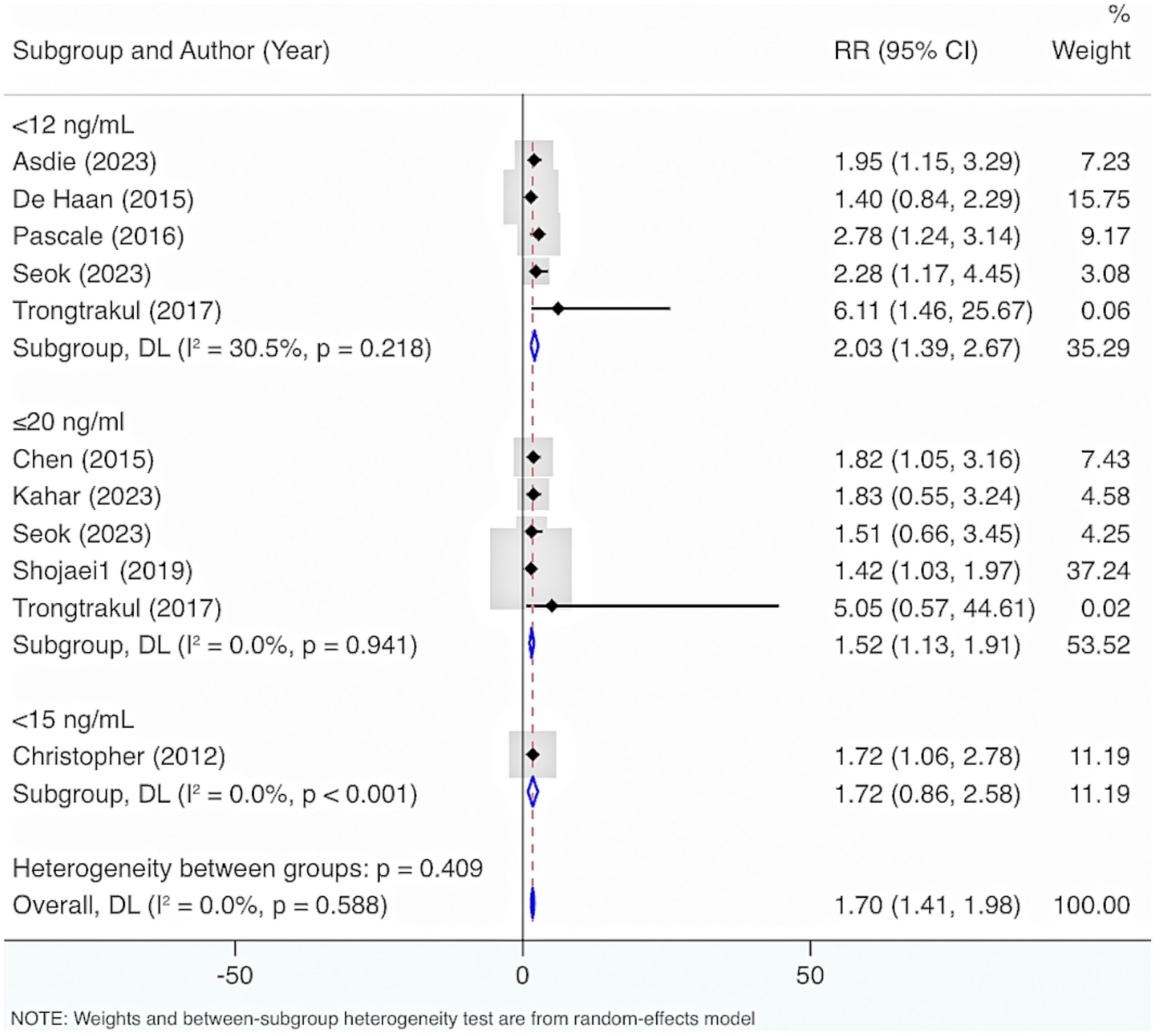
Subgroup analysis grounded in levels of VD deficiency.

Nine studies were included. The results from the Cochrane *Q* test (*p* = 0.49) and *I*^2^ estimate (0%) showed no significant heterogeneity. Meta-analysis leveraging a fixed-effects model displayed that deficiency of VD elevated the risk of mortality (RR = 1.67, 95% CI: 1.38, 1.97, *p* < 0.001) (Figure 12). Further subgroup analysis grounded in levels of VD deficiency illustrated that a more severe deficiency in VD was linked to a higher risk of mortality. When VD levels were <12 ng/mL, the likelihood of mortality was highest (RR = 2.03, 95% CI: 1.39, 2.67, *p* < 0.001) (Figure 13).

#### 3.5.7 VD supplementation and length of ICU stay

Four studies were incorporated. Results from the Cochrane *Q* test (*p* < 0.001) and I^2^ estimate (89.9%) showed considerable heterogeneity. Meta-analysis leveraging a random-effects model displayed that supplementing VD did not shorten the length of ICU stay (MD = −0.11, 95% CI: −0.62, 0.40, *p* = 0.67) (Supplementary Figure S3).

#### 3.5.8 Predictive value of VD levels for mortality

Three studies were included. The combined sensitivity and specificity were 0.81 (95% CI: 0.73, 0.87) and 0.31 (95% CI: 0.25, 0.39). In terms of sensitivity, results from the Cochrane *Q* test (*p* = 0.07) and *I*^2^ estimate (62%) showed significant heterogeneity. For specificity, results from the Cochrane *Q* test (*p* = 0.001) and *I*^2^ estimate (85%) showed significant heterogeneity (Table 2).

**Table 2:** Predictive value of VD levels for adult sepsis mortality.

Study	TP	FP	FN	TN	Sensitivity (95% CI)	Specificity (95% CI)
Nguyen 2013	8	66	2	15	0.80(0.44–0.98)	0.19(0.11–0.29)
Shojaei 2019	90	33	16	29	0.85(0.77–0.91)	0.47(0.34–0.60)
Tosoni 2021	13	28	8	14	0.62(0.38–0.82)	0.33(0.20–0.50)

### 3.6 Sensitivity analysis and publication bias

Sensitivity analysis was implemented on such outcomes as levels of VD and prevalence of VD deficiency and insufficiency among neonates and pediatric sepsis patients. The leave-one-out method results displayed that excluding any individual research did not substantially affect the main conclusions (Supplementary Figures S4, S5). Sensitivity analysis was implemented on such outcomes as levels of VD, prevalence of VD deficiency and insufficiency among adult sepsis patients, VD levels and the risk of sepsis mortality based on binary variables, and deficiency of VD and mortality of sepsis through multivariable statistical analysis. The results illustrated that excluding any individual research did not substantially affect the main conclusions (Supplementary Figures S6–S9).

Publication bias was estimated for such outcomes as levels of VD and prevalence of VD deficiency and insufficiency among adult sepsis individuals. The funnel plot showed an asymmetric distribution in the analysis of VD levels (Supplementary Figure S10). Egger’s test illustrated potential publication bias (*p* = 0.03). Furthermore, the trim-and-fill analysis indicated that no missing studies needed to be imputed. This suggests that no funnel plot asymmetry attributable to small-study effects was detected in our analysis, thereby supporting the robustness of the current meta-analytic results (Supplementary Figure S11). The funnel plot also showed an asymmetric distribution in the analysis of the prevalence of VD deficiency and insufficiency (Supplementary Figure S12). Egger’s test illustrated potential publication bias (*p* < 0.05). Since applying the trim-and-fill method directly to raw proportions can yield illogical results (e.g., values >1), we first performed a logit transformation on the proportions from the original data and conducted the trim-and-fill analysis on the transformed scale. After imputing 10 theoretically missing studies, the pooled effect size was 22.9% (95% CI, 9.3, 56.3%, *p* = 0.001). This estimate, which is slightly lower than the initial pooled result, suggests that the original findings may have been influenced to some extent by publication bias (Supplementary Figure S13).

## 4 Discussion

This study is pioneering in comprehensively estimating the VD levels, prevalence of deficiency, interrelation of VD levels with incidence and mortality of sepsis, and forecasting capability of VD among pediatric and adult individuals with sepsis separately. This assessment is conducted in alignment with guidelines on early detection, appropriate intervention, and prognostic value.

This research illustrated that the VD status was relatively deficient among neonates and pediatric individuals with sepsis. The overall prevalence of VD deficiency and insufficiency reached 54%. This result aligns with prior findings (12, 63). Xiao et al. (12) observed that, relative to the control group of healthy individuals, neonates and children suffering from sepsis had decreased VD levels. Yu et al. (63) reported that, compared to neonates and children without sepsis, critically ill infants and children suffering from sepsis had decreased VD levels and more severe deficiency. Workneh Bitew et al. (78) found that the overall prevalence of VD deficiency in neonates with sepsis reached 79.4%. The prevalence of VD deficiency found in our study is relatively lower than that of Workneh Bitew et al. (78). The disparity may arise from differences in the years of publication of the studies included, as most studies we included were published after 2019 (accounting for 78% of the total studies). Improvements in medical care, along with increased awareness of supplementing VD among physicians and parents may induce a reduction in the prevalence of VD deficiency and insufficiency. Additionally, this research illustrated a considerable interrelation between deficiency of VD and mortality among neonates and pediatric sepsis patients. However, previous meta-analyses regarding neonates and pediatric sepsis populations did not report this finding (63, 64). The interrelation between VD deficiency and mortality of neonates and children has been confirmed in other studies. He et al. (65) indicated an independent interrelation between deficiency of VD in children and scores of pediatric mortality risk III. Su and Jia (66) indicated that children with VD deficiency had a significantly elevated likelihood of developing acute mortality and critical mortality relative to those with normal levels of VD, which was 1.77 times that of the normal VD group. This may be due to that VD deficiency could increase the likelihood of respiratory failure and heart failure, thereby increasing mortality (67, 68). Therefore, early identification is recommended for neonates and pediatric sepsis patients with VD deficiency to respond to adverse outcomes in time. Finally, due to the limited number of included studies involving neonatal and pediatric patients with sepsis (*n* = 10), and the presence of geographically imbalanced distribution (India = 6, Turkey = 2, Egypt = 1, Iran = 1), the interpretation of these findings should be approached with caution, and their generalizability to other regions requires careful consideration.

In adult sepsis patients, VD status was notably deficient, with the overall prevalence of VD deficiency and insufficiency reaching 55%. VD is known for its regulatory function in the immune system and its potential in infection prevention (69, 70). Consequently, in sepsis patients, VD levels are relatively low and the prevalence of deficiency and insufficiency is relatively high. Furthermore, this study observed a considerable interrelation between the deficiency of VD and both the incidence and mortality of sepsis (*p* < 0.001). Supplementing VD can considerably diminish the risk of mortality (*p* < 0.05). As an essential micronutrient, VD is crucial in the pathogenesis and mortality of sepsis. VD exerts its effects by binding to VD receptors expressed on T lymphocytes, B lymphocytes, macrophages, and dendritic cells. It specifically modulates both innate and adaptive immune responses, which is vital to maintain immune homeostasis among sepsis patients. Meanwhile, it can prevent severe disease progression and influence prognosis (71, 72). For sepsis patients who are continually challenged by pathogens and uncontrolled immune responses, supplementing VD may be the optimal therapeutic intervention to prevent adverse outcomes. On one hand, VD may restore the levels of serum IL-37, thereby enhancing antimicrobial activity (73); on the other hand, it regulates innate immunity to protect the body from excessive production of inflammatory cytokines (74). Since this outcome was based on only four studies, only one of which was a high-quality RCT, the results may not sufficiently verify the therapeutic effect of vitamin D supplementation on mortality in sepsis patients. This may be attributed to the fact that research in this area is still in its early stages. Despite our comprehensive search strategy, few studies met the eligibility criteria. Furthermore, as the available studies did not provide sufficient intervention details, it was not possible to perform subgroup analyses based on vitamin D dosage, treatment duration, or route of administration. Therefore, future randomized controlled trials should focus on investigating the dose–response relationship of vitamin D supplementation on outcomes in sepsis patients, in order to provide a scientific basis for developing standardized clinical intervention protocols. Lastly, this study also observed that VD had a reliable forecasting ability for mortality in sepsis patients. A recent meta-analysis of multivariable-adjusted follow-up studies has confirmed this finding, showing that severe VD deficiency is independently correlated with an elevated likelihood of mortality in adult individuals with sepsis (11). This may be due to the interaction between VD receptors and related signaling pathways, which enables VD to help maintain the fundamental functions of the heart (75), lungs (76), and kidneys (77) during severe infections.

### 4.1 Study limitations

Our study has several limitations. First, although most eligible studies were cohort studies, some case–control and cross-sectional studies were also included. Future research could focus exclusively on cohort studies to offer reliable evidence on the long-term effects of VD in individuals with sepsis. Second, there was high heterogeneity in several outcome indicators. Despite conducting subgroup analyses, the sources of heterogeneity were not identified. This may be related to the potential influence of several research characteristics on the outcomes, for instance, the nutritional status of the patients, diagnostic criteria for sepsis, the critical threshold for deficiency of VD, the dose of VD supplementation, and the units of VD measurement. Future research should incorporate more studies and conduct meta-regression to figure out the sources of heterogeneity. Third, this study merged the results of adjusted and unadjusted multivariate analyses, which could make the findings susceptible to residual bias and unadjusted confounding factors. Hence, the results need to be interpreted cautiously. Fourth, the use of a uniform vitamin D deficiency threshold across all pediatric age groups, without distinguishing between neonates and other subgroups, represents another limitation. Given that neonates may have distinct vitamin D metabolic profiles and risk factors, applying a single cut-off value may not accurately reflect the status of all populations. This could be a source of potential heterogeneity and may limit the extension of our conclusions to specific age subgroups. Future studies are urgently needed to establish and validate age-specific thresholds. Fifth, given the well-established physiological differences in vitamin D metabolism between neonates and children, pooling these populations may introduce substantial heterogeneity and could obscure distinct associations within each subgroup. Therefore, the pooled effect estimates should be interpreted with caution, and future studies should report outcomes stratified by these key age groups. Lastly, currently, no research investigates the predictive value of VD for the incidence or mortality of neonatal and individuals with pediatric sepsis. Future studies could focus on this area, preventing adverse outcomes.

## 5 Conclusion

VD status is deficient among both adult and pediatric sepsis patients, with a high prevalence of deficiency and insufficiency. VD deficiency is significantly linked to mortality in pediatric sepsis. Early supplementation of VD is recommended to prevent adverse outcomes. In adults with sepsis, VD deficiency is tightly linked to both the incidence and mortality. VD supplementation considerably reduces the risk of mortality. However, further large-scale RCTs are required to validate the therapeutic capability of supplementing VD. Interpretation of the results regarding neonatal and pediatric sepsis patients should be made with caution, and their generalizability to other regions requires careful consideration. VD can be a valuable biomarker to forecast mortality in adults with sepsis. Nevertheless, the data in the existing literature are not sufficient to reliably estimate its accuracy. Additional prospective studies are necessitated.

## Data Availability

The original contributions presented in the study are included in the article/Supplementary material. Further inquiries can be directed to the corresponding authors.

## Footnotes

^1^ https://www.crd.york.ac.uk/PROSPERO/home
