# Supplementary material for: Assessment of the influence of vitamin D in patients with sepsis: a systematic review and meta-analysis
Source: Front Nutr. 2025 Oct 13;12:1670083. doi: 10.3389/fnut.2025.1670083 (PMC12554583; doi:10.3389/fnut.2025.1670083)
Supplement: Supplementary file 1 [file Table_1.docx]

**Table S1** Search strategy

**Cochrane**

ID Search

ID Search Hits

1 MeSH descriptor: [Vitamin D] explode all trees 7592

#2 MeSH descriptor: [Sepsis] explode all trees 6480

#3 (vd or vitamin D or hydroxycholecalciferols or cholecalciferol or ergocalciferols or 25 hydroxyvitamin d or dihydrotachysterol or calciol or calcifediol or vitamin d3 or cholecalciferols or vitamin d deficiency or 25(oh)d or hydroxyvitamin d):ti,ab,kw 21252

#4 (bloodstream infections or pyemia or pyemias or pyohemia or pyohemias or pyaemia or pyaemias or septicemia or septicemias or blood poisoning or blood poisonings or severe sepsis or septic disease or sepsis or bloodstream infection):ti,ab,kw 18001

#5 #1 or #3 21845

#6 #2 or #4 20414

#7 #5 and #6 188

**Pubmed**

| Search number | Search Details | Results |
| --- | --- | --- |
| 5:4 and 3 | (("Vitamin D"[Mesh]) OR ((((((((((((((vitamin D[Title/Abstract]) OR (vd[Title/Abstract])) OR (Cholecalciferol[Title/Abstract])) OR (Hydroxycholecalciferols[Title/Abstract])) OR (Ergocalciferols[Title/Abstract])) OR (25-Hydroxyvitamin D[Title/Abstract])) OR (Dihydrotachysterol[Title/Abstract])) OR (Calciol[Title/Abstract])) OR (calcifediol[Title/Abstract])) OR (Vitamin D3[Title/Abstract])) OR (Cholecalciferols[Title/Abstract])) OR (Vitamin D deficiency[Title/Abstract])) OR (25(OH)D[Title/Abstract])) OR (Hydroxyvitamin D[Title/Abstract]))) AND (("Sepsis"[Mesh]) OR (((((((((((((((sepsis[Title/Abstract]) OR (Bloodstream Infection)) OR (Bloodstream Infections)) OR (Pyemia)) OR (Pyemias)) OR (Pyohemia)) OR (Pyohemias)) OR (Pyaemia)) OR (Pyaemias)) OR (Septicemia)) OR (Septicemias)) OR (Blood Poisoning)) OR (Blood Poisonings)) OR (Severe Sepsis)) OR (septic disease))) | 872 |
| 4 | ("Sepsis"[Mesh]) OR (((((((((((((((sepsis[Title/Abstract]) OR (Bloodstream Infection)) OR (Bloodstream Infections)) OR (Pyemia)) OR (Pyemias)) OR (Pyohemia)) OR (Pyohemias)) OR (Pyaemia)) OR (Pyaemias)) OR (Septicemia)) OR (Septicemias)) OR (Blood Poisoning)) OR (Blood Poisonings)) OR (Severe Sepsis)) OR (septic disease)) | 321122 |
| 3:1 or 2 | ("Vitamin D"[Mesh]) OR ((((((((((((((vitamin D[Title/Abstract]) OR (vd[Title/Abstract])) OR (Cholecalciferol[Title/Abstract])) OR (Hydroxycholecalciferols[Title/Abstract])) OR (Ergocalciferols[Title/Abstract])) OR (25-Hydroxyvitamin D[Title/Abstract])) OR (Dihydrotachysterol[Title/Abstract])) OR (Calciol[Title/Abstract])) OR (calcifediol[Title/Abstract])) OR (Vitamin D3[Title/Abstract])) OR (Cholecalciferols[Title/Abstract])) OR (Vitamin D deficiency[Title/Abstract])) OR (25(OH)D[Title/Abstract])) OR (Hydroxyvitamin D[Title/Abstract])) | 120776 |
| 2 | (((((((((((((vitamin D[Title/Abstract]) OR (vd[Title/Abstract])) OR (Cholecalciferol[Title/Abstract])) OR (Hydroxycholecalciferols[Title/Abstract])) OR (Ergocalciferols[Title/Abstract])) OR (25-Hydroxyvitamin D[Title/Abstract])) OR (Dihydrotachysterol[Title/Abstract])) OR (Calciol[Title/Abstract])) OR (calcifediol[Title/Abstract])) OR (Vitamin D3[Title/Abstract])) OR (Cholecalciferols[Title/Abstract])) OR (Vitamin D deficiency[Title/Abstract])) OR (25(OH)D[Title/Abstract])) OR (Hydroxyvitamin D[Title/Abstract]) | 104184 |
| 1 | "Vitamin D"[Mesh] | 72089 |

**Embase**

| No. | Query | Results |
| --- | --- | --- |
| #7 | #5 AND #6 | 1724 |
| #6 | #3 OR #4 | 437692 |
| #5 | #1 OR #2 | 202587 |
| #4 | 'bloodstream infections':ab,ti OR pyemia:ab,ti OR pyemias:ab,ti OR pyohemia:ab,ti OR pyohemias:ab,ti OR pyaemia:ab,ti OR pyaemias:ab,ti OR septicemia:ab,ti OR septicemias:ab,ti OR 'blood poisoning':ab,ti OR 'blood poisonings':ab,ti OR 'severe sepsis':ab,ti OR 'septic disease':ab,ti OR sepsis:ab,ti | 234593 |
| #3 | 'sepsis'/exp | 379983 |
| #2 | 'vitamin d'/exp | 197967 |
| #1 | ((((((vd:ab,ti OR vitamin:ab,ti) AND d:ab,ti OR hydroxycholecalciferols:ab,ti OR cholecalciferol:ab,ti OR ergocalciferols:ab,ti OR '25 hydroxyvitamin':ab,ti) AND d:ab,ti OR dihydrotachysterol:ab,ti OR calciol:ab,ti OR calcifediol:ab,ti OR vitamin:ab,ti) AND d3:ab,ti OR cholecalciferols:ab,ti OR vitamin:ab,ti) AND d:ab,ti AND deficiency:ab,ti OR 25:ab,ti) AND oh:ab,ti AND d:ab,ti OR hydroxyvitamin:ab,ti) AND d:ab,ti | 53226 |

**Web of Science**

1: vitamin D (Topic) OR vd (Topic) OR Cholecalciferol (Topic) OR Hydroxycholecalciferols (Topic) OR Ergocalciferols (Topic) OR 25-Hydroxyvitamin D (Topic) OR Dihydrotachysterol (Topic) OR Calciol (Topic) OR calcifediol (Topic) OR Vitamin D3 (Topic) OR Cholecalciferols (Topic) OR 25(OH)D (Topic) OR Hydroxyvitamin D (Topic) Results: 901

2: sepsis (Topic) OR Bloodstream Infection (Topic) OR Bloodstream Infections (Topic) OR Pyemia (Topic) OR Pyemias (Topic) OR Pyohemia (Topic) OR Pyohemias (Topic) OR Pyaemia (Topic) OR Pyaemias (Topic) OR Septicemia (Topic) OR Septicemias (Topic) OR Blood Poisoning (Topic) OR Blood Poisonings (Topic) OR Severe Sepsis (Topic) OR septic disease (Topic) Results: 12654

3: #2 AND #1 Results: 106

**Table S2** NOS quality score included in the cohort study

| Study | Selection of research population | | | | Comparability of cohorts on the basis of the design or analysis | Outcome measure | | | Summary |
| --- | --- | --- | --- | --- | --- | --- | --- | --- | --- |
|  | **Representativeness of the exposed cohort** | **Selection of the non-exposed cohort** | **Ascertainment of exposure** | **Demonstration that outcome of interest (dementia) was not present at start of study** |  | **Assessment of outcome** | **Was follow-up long enough for outcomes to occur?** | **Adequacy of follow up of cohorts** |  |
| Alves2015 | 1 | 1 | 1 | 1 | 1 | 1 | 1 | 1 | 8 |
| Asdie2023 | 1 | 1 | 1 | 1 | 1 | 1 | 1 | 1 | 8 |
| Aydemir2014 | 1 | 1 | 1 | 1 | 0 | 1 | 1 | 1 | 7 |
| Chen2015 | 1 | 1 | 1 | 1 | 1 | 1 | 1 | 1 | 8 |
| Pascale2016 | 1 | 1 | 1 | 1 | 1 | 1 | 1 | 1 | 8 |
| Greulich2017 | 1 | 1 | 1 | 1 | 0 | 1 | 0 | 1 | 6 |
| Guan2023 | 1 | 1 | 1 | 1 | 2 | 1 | 1 | 1 | 9 |
| GUL2019 | 1 | 1 | 1 | 1 | 0 | 1 | 1 | 1 | 8 |
| Jovanovich2014 | 1 | 1 | 1 | 1 | 2 | 1 | 0 | 1 | 8 |
| Kahar2023 | 1 | 1 | 0 | 1 | 1 | 1 | 0 | 1 | 6 |
| Kempker2012 | 1 | 1 | 0 | 1 | 0 | 1 | 0 | 1 | 5 |
| Kumar2020 | 1 | 1 | 1 | 1 | 1 | 1 | 0 | 1 | 7 |
| Nguyen2013 | 1 | 1 | 1 | 1 | 2 | 1 | 0 | 1 | 8 |
| Ozdemir2019 | 1 | 1 | 0 | 1 | 0 | 1 | 0 | 1 | 5 |
| Ratzinger2017 | 1 | 1 | 1 | 1 | 1 | 1 | 0 | 1 | 7 |
| Singh2020 | 1 | 1 | 1 | 1 | 0 | 1 | 0 | 1 | 6 |
| Tosoni2021 | 1 | 1 | 1 | 1 | 1 | 1 | 0 | 1 | 7 |
| Trongtrakul2017 | 1 | 1 | 1 | 1 | 2 | 1 | 1 | 1 | 9 |
| Seok2023 | 1 | 1 | 1 | 1 | 2 | 1 | 0 | 1 | 8 |
| Yang2023 | 1 | 1 | 1 | 1 | 2 | 1 | 0 | 1 | 8 |
| Alves2013 | 1 | 1 | 1 | 1 | 0 | 1 | 0 | 0 | 5 |
| Anis2018 | 1 | 1 | 1 | 1 | 0 | 1 | 1 | 0 | 6 |
| Christopher2012 | 1 | 1 | 1 | 1 | 2 | 1 | 1 | 0 | 8 |
| De Haan2015 | 1 | 1 | 1 | 1 | 0 | 1 | 1 | 0 | 6 |
| Pinargote2018 | 1 | 1 | 1 | 1 | 2 | 1 | 1 | 0 | 8 |
| Salciccioli2012 | 1 | 1 | 1 | 1 | 2 | 1 | 1 | 0 | 8 |
| Satheesh2013 | 1 | 1 | 1 | 1 | 0 | 1 | 1 | 0 | 6 |
| Yoo2020 | 1 | 1 | 1 | 1 | 2 | 1 | 1 | 1 | 9 |
| Li2025 | 1 | 1 | 1 | 1 | 2 | 1 | 1 | 1 | 9 |

**Table S3** NOS quality score included in the case control study

| Study | Selection of cases and controls | | | | Comparability between groups | Exposure factors | | | Summary |
| --- | --- | --- | --- | --- | --- | --- | --- | --- | --- |
|  | **Clear case definition** | **Representativeness of the case group** | **Comparison selection** | **Definition of control group** |  | **exposure determination** | **Same method** | **No response rate** |  |
| Agrawal2019 | 1 | 1 | 1 | 1 | 0 | 1 | 1 | 0 | 6 |
| Gamal2017 | 1 | 1 | 1 | 1 | 0 | 1 | 1 | 0 | 6 |
| Kubsad2021 | 1 | 1 | 1 | 1 | 0 | 1 | 1 | 0 | 6 |
| Mohapatra2018 | 1 | 1 | 1 | 1 | 0 | 1 | 1 | 1 | 7 |
| Prasad2018 | 1 | 1 | 1 | 1 | 0 | 1 | 1 | 0 | **6** |

**Table S4** AHRQ quality score included in cross-sectional studies

| **Study** | **v1** | **v2** | **v3** | **v4** | **v5** | **v6** | **v7** | **v8** | **v9** | **v10** | **v11** | **Summary** |
| --- | --- | --- | --- | --- | --- | --- | --- | --- | --- | --- | --- | --- |
| Vanichkulbodee2023 | Yes | Yes | Yes | Yes | No | Yes | No | Yes | Yes | Yes | Yes | 9 |
| Shojaei2019 | Yes | Yes | Yes | Yes | No | Yes | No | Yes | No | No | No | 6 |
| Zakerihamidi2023 | Yes | Yes | Yes | Yes | No | Yes | No | Yes | No | No | No | 6 |
| Romposra2020 | Yes | Yes | Yes | Yes | No | Yes | No | Yes | Yes | Yes | No | 8 |

**
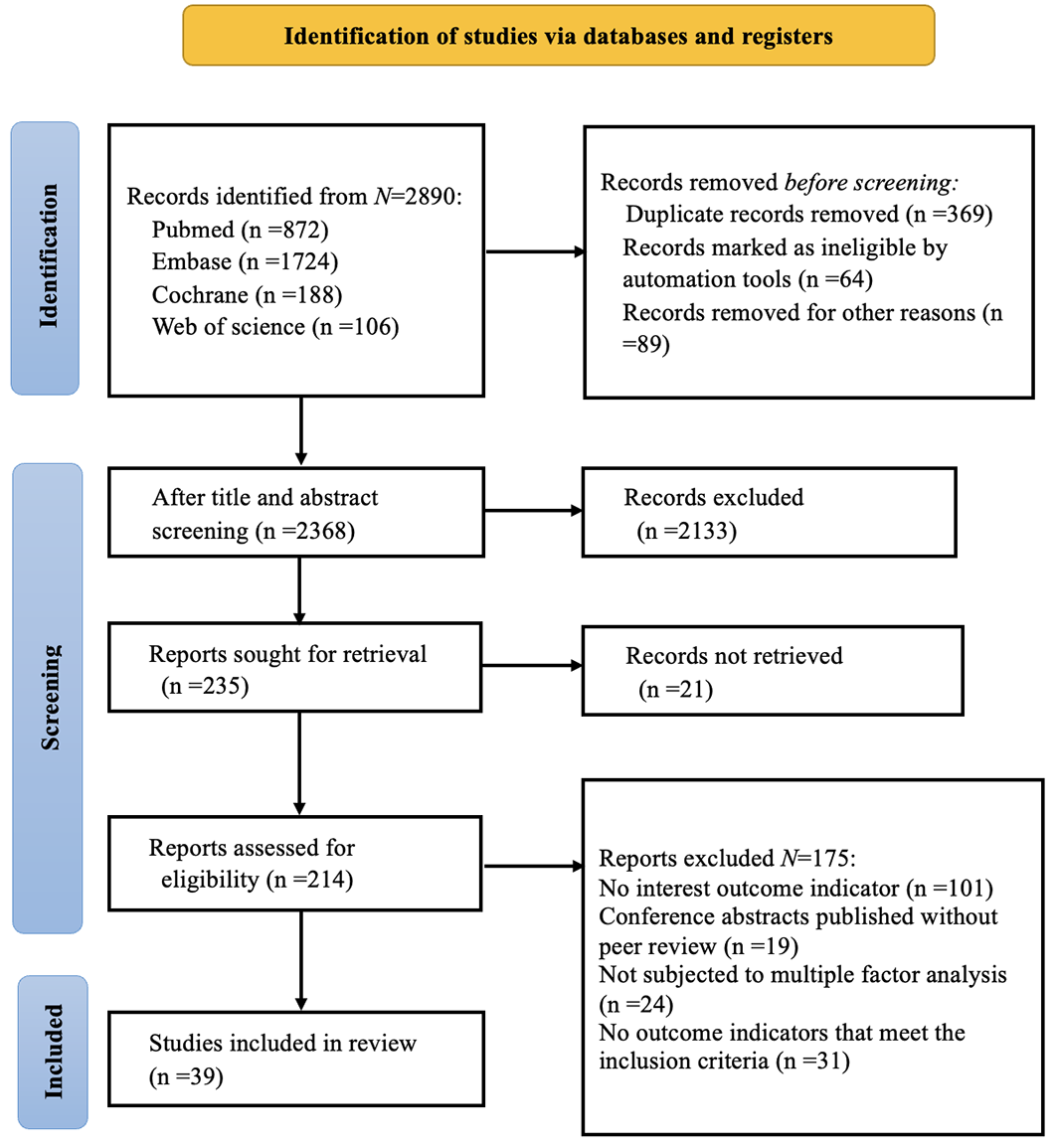
**

**Figure S1** Literature screening process.


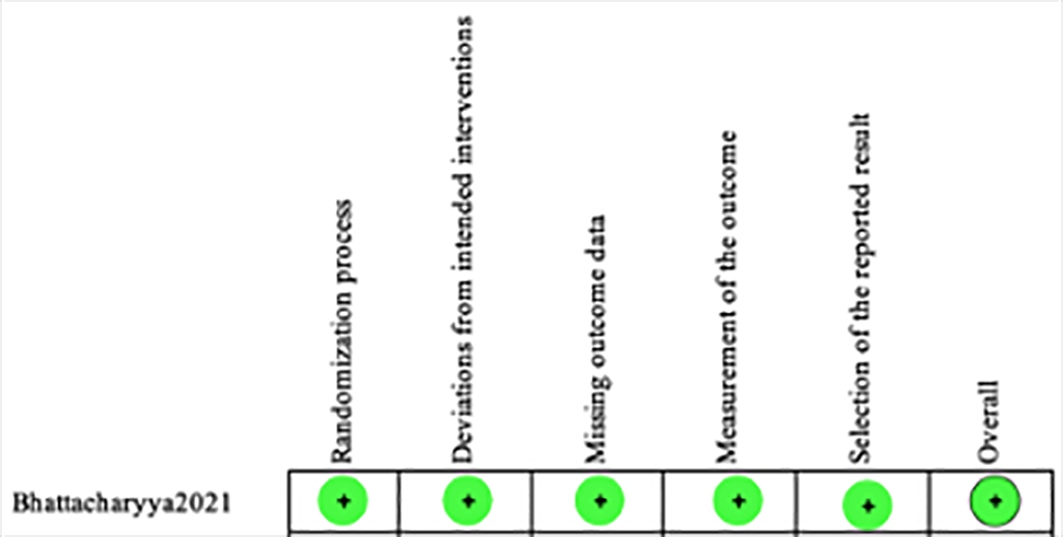


**Figure S2** Summary of the risk of bias.


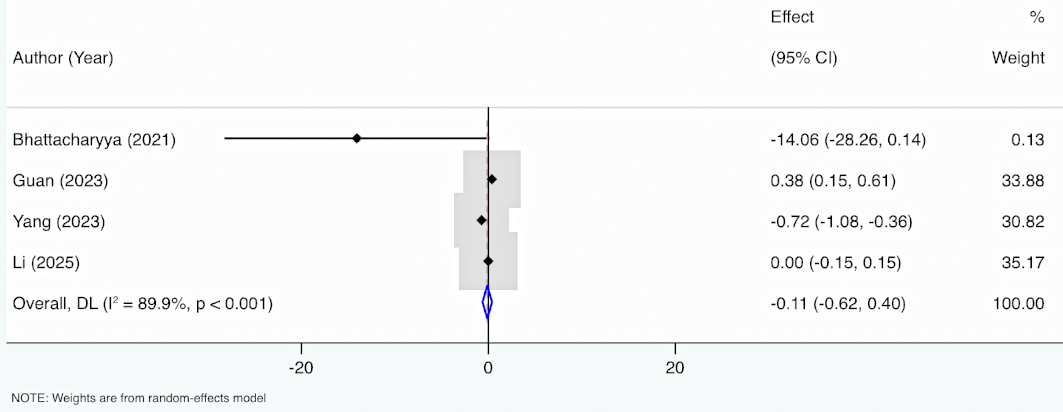


**Figure S3** The relationship between VD supplementation and length of ICU stay in adult sepsis patients.


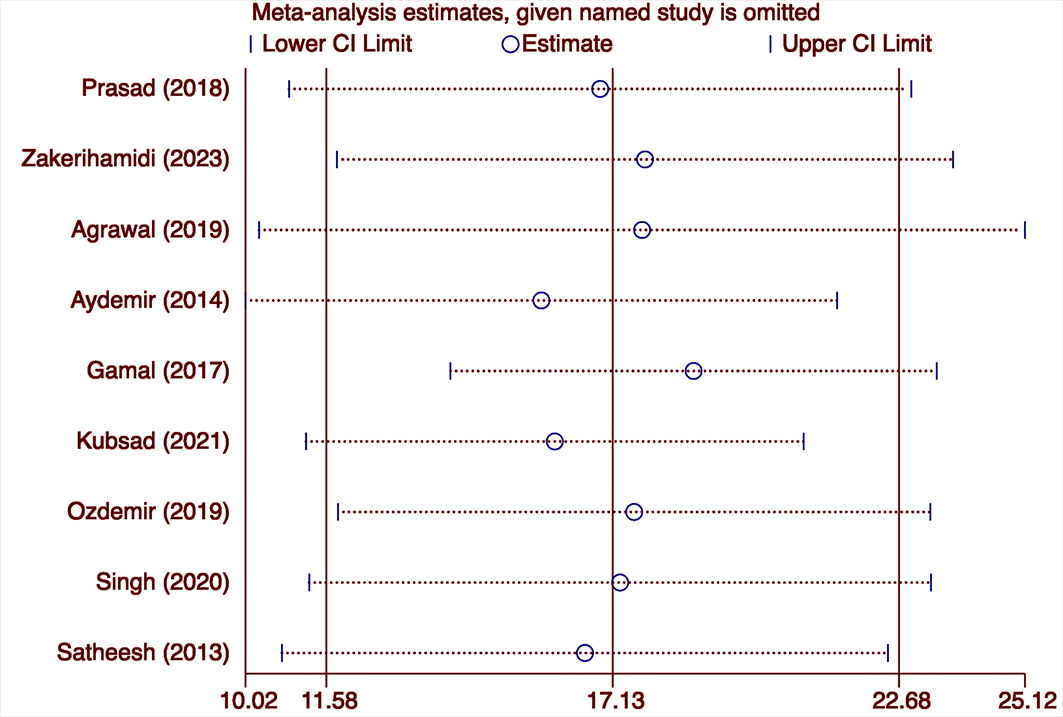


**Figures S4** Sensitivity analysis of VD levels in neonatal and pediatric sepsis patients.


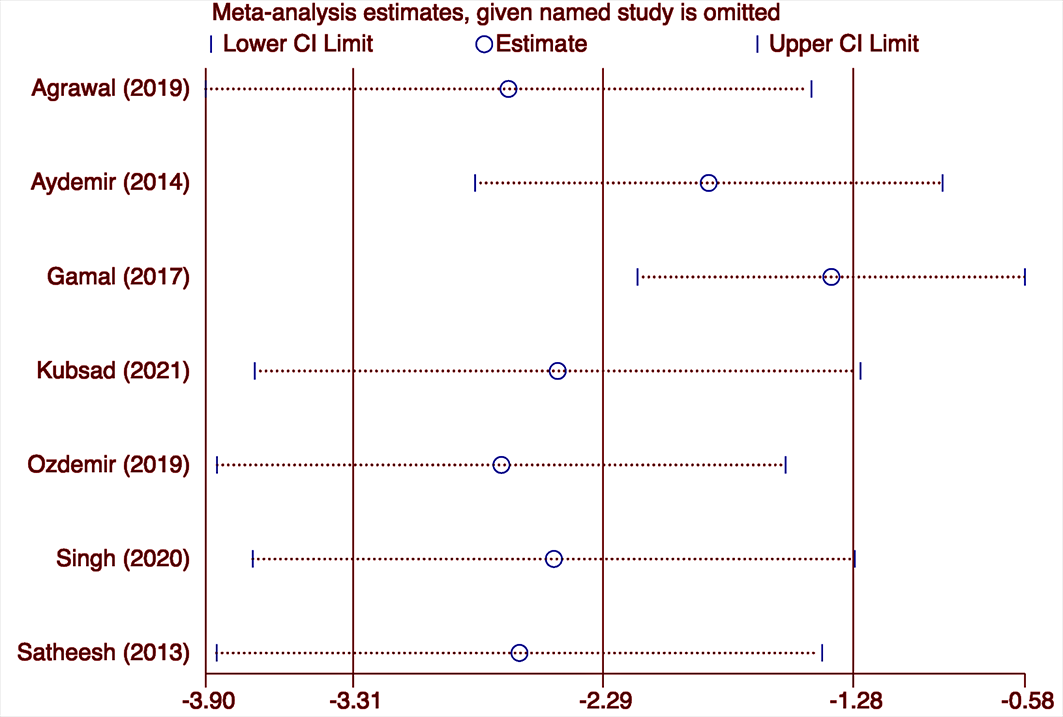


**Figures S5** Sensitivity analysis of VD deficiency and insufficiency in neonatal and pediatric sepsis patients.


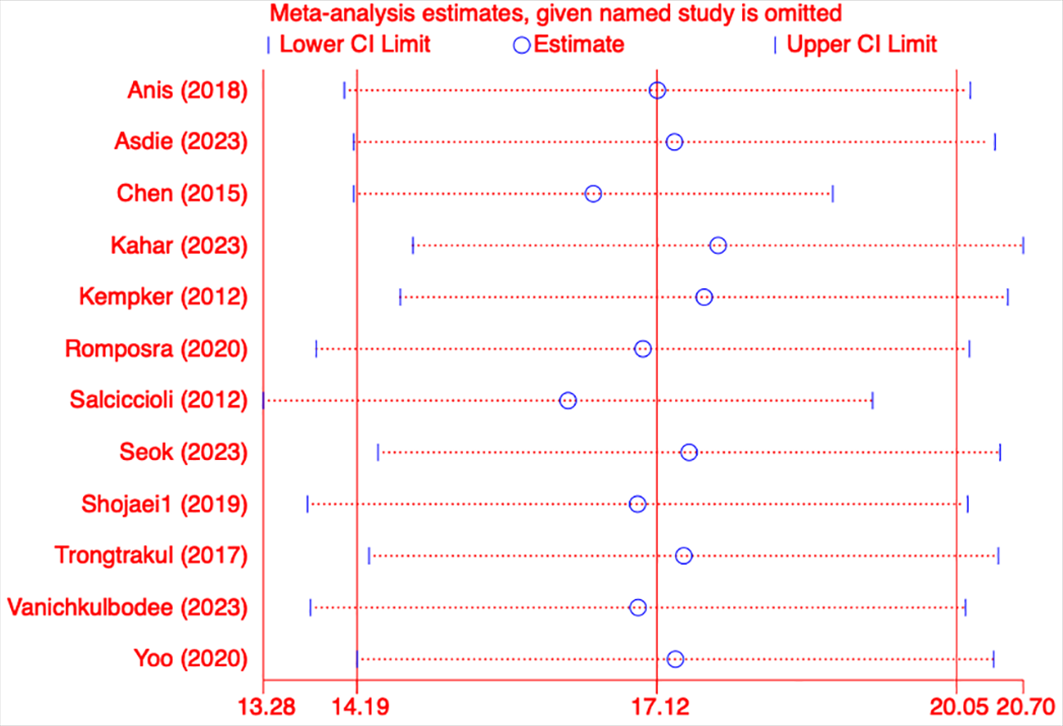


**Figures S6** Sensitivity analysis of VD levels in adult sepsis patients.


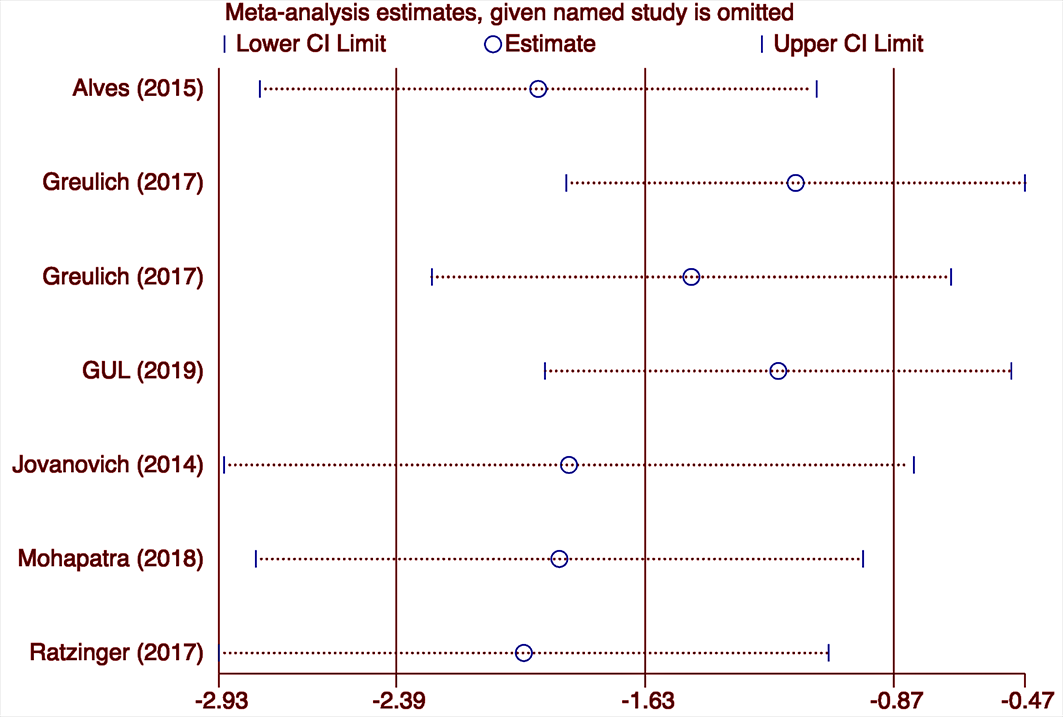


**Figures S7** Sensitivity analysis of the prevalence of VD deficiency and insufficiency in adult sepsis patients.


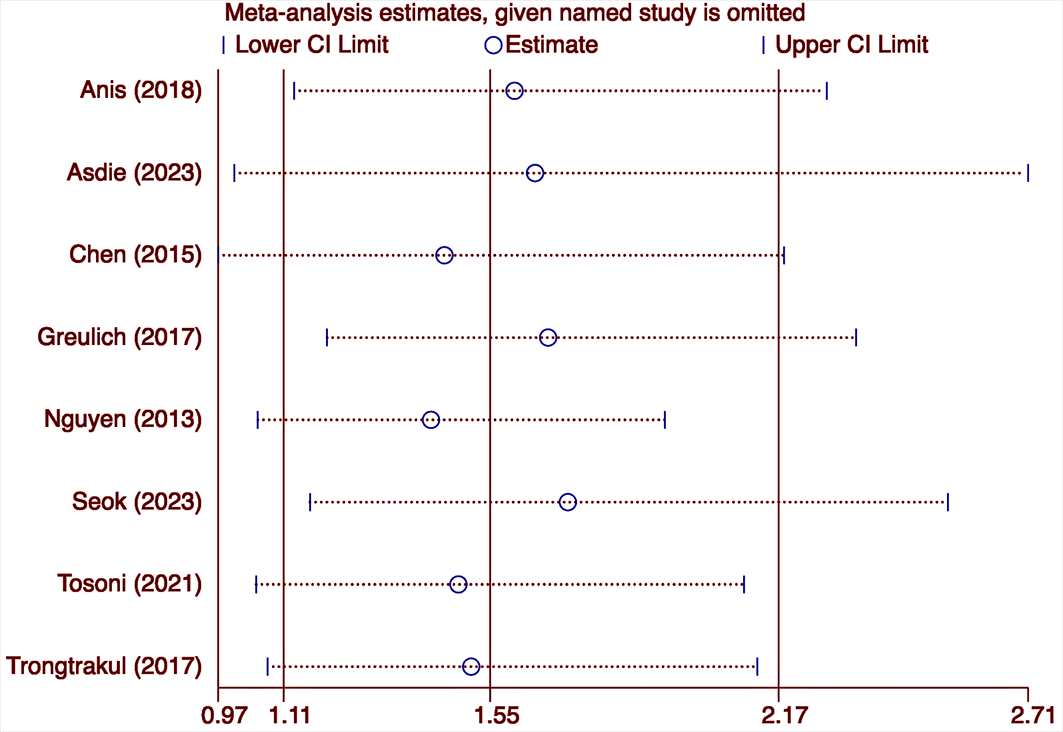


**Figures S8** Sensitivity analysis of VD levels and mortality risk in adult sepsis patients.


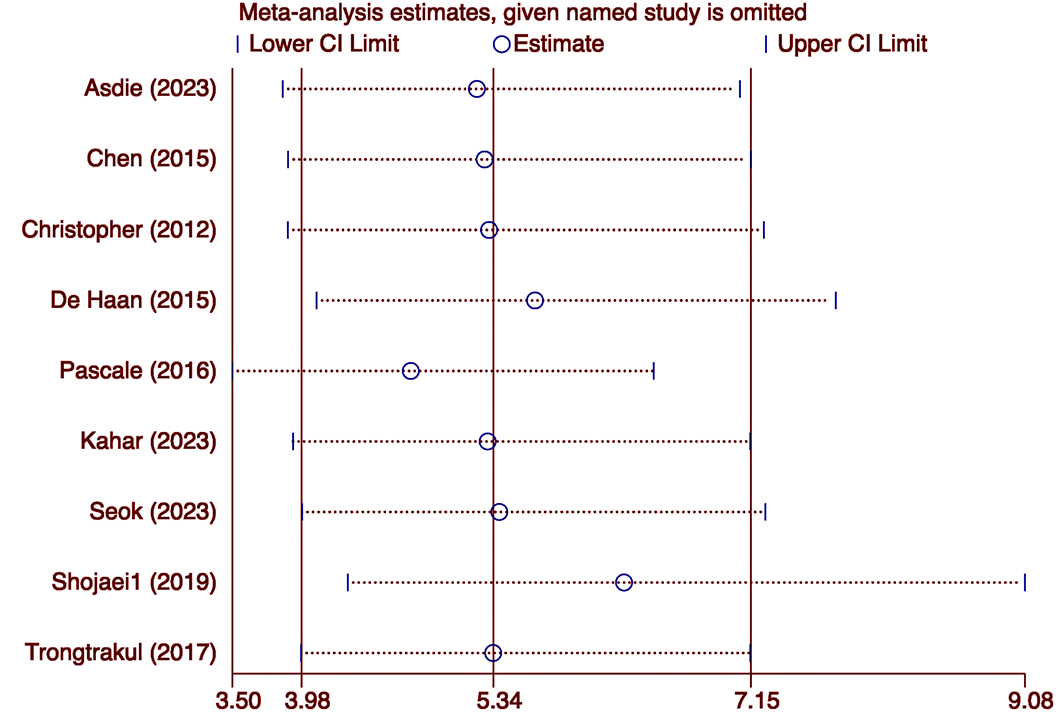


**Figures S9** Sensitivity analysis of VD deficiency and mortality risk in adult sepsis patients.


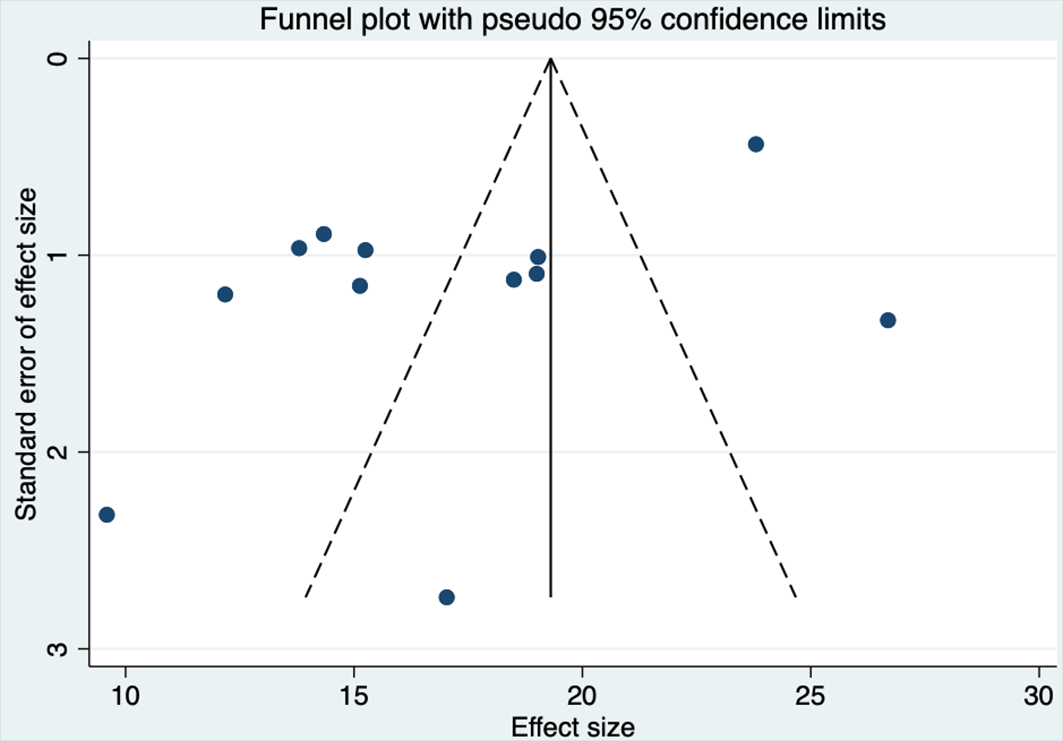


**Figure S10** Funnel plot of VD levels in adult sepsis patients.

**
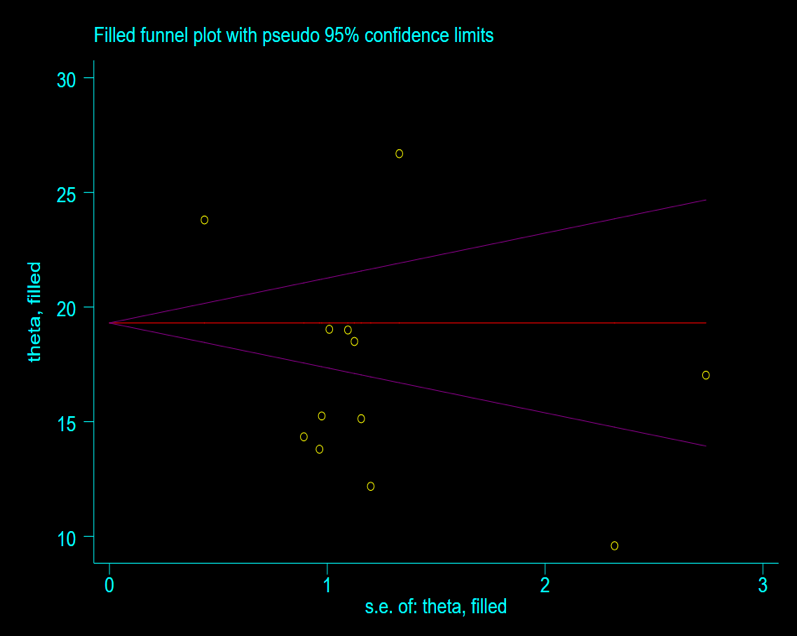
**

**Figure S11** The trim-and-fill method of VD levels in adult sepsis patients.


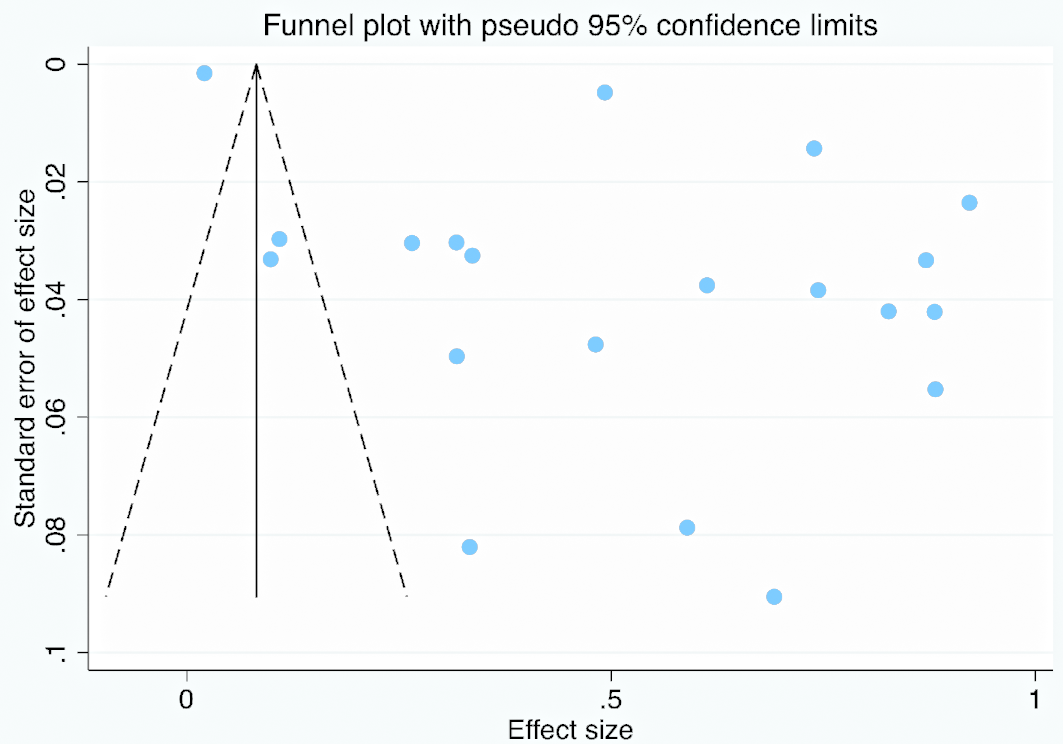


**Figure S12** Funnel plot of the prevalence of VD deficiency and insufficiency in adult sepsis patients.


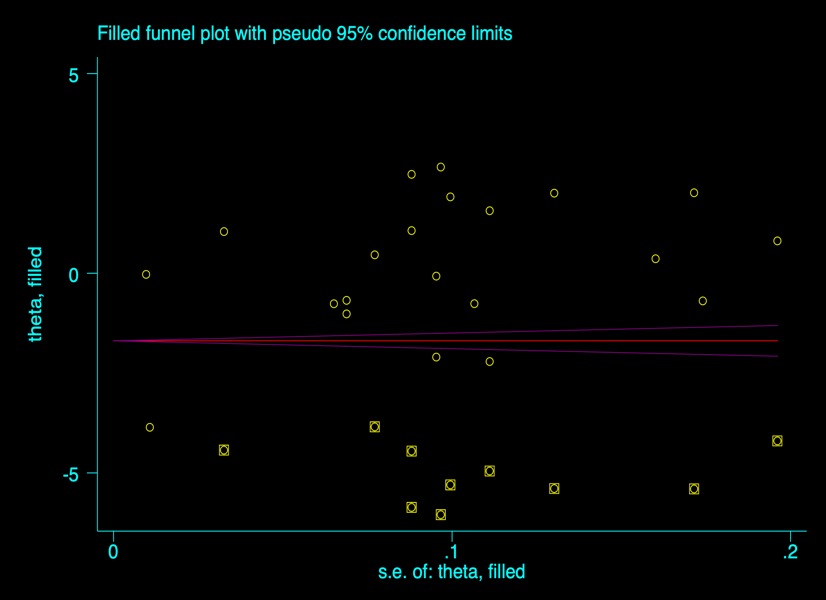


**Figure S13** The trim-and-fill method of the prevalence of VD deficiency and insufficiency in adult sepsis patients.
